# Polyphenols from Byproducts: Their Applications and Health Effects

**DOI:** 10.3390/antiox15010087

**Published:** 2026-01-09

**Authors:** Ranya Demir, Sümeyye Sarıtaş, Mikhael Bechelany, Sercan Karav

**Affiliations:** 1 Department of Molecular Biology and Genetics, Çanakkale Onsekiz Mart University, Çanakkale 17000, Türkiye; ranyaddemir@gmail.com (R.D.); sumeyyesaritas@stu.comu.edu.tr (S.S.); 2 European Institute for Membranes (IEM), UMR-5635, University of Montpellier, ENSCM, CNRS, Place Eugène Bataillon, CEDEX 5, F-34095 Montpellier, France

**Keywords:** agricultural biomass, sustainability, secondary metabolites, antiviral, antimicrobial, antioxidant, eco-friendly, green method

## Abstract

Plant byproducts represent a valuable and underutilized source of bioactive compounds. Among these, phenolic compounds have attracted growing interest from the agricultural, cosmetic, and food industries due to their diverse biological activities. These naturally occurring compounds are derived from various plant species, and they exhibit strong antioxidant, antimicrobial, and antiviral properties. Their yield, as well as quality and bioavailability, has improved with more recent advancements within green extraction, as well as purification and characterization techniques. Several phenolic compounds exhibit strong antiviral and antioxidant activities, which are highlighting their value as bioactive compounds. It is essential to evaluate extraction methods for high-yield phenolic compounds from plant byproducts so that they can contribute to the circular bioeconomy, reduction in environmental waste, and development of biomedical and food industrial applications. Their physicochemical characteristics and potential applications may lead to a determination by contributing to promising fields through expanded in vitro, in vivo, and in silico experiments. This review summarizes current research on the extraction, recovery, and applications of phenolic compounds derived from plant byproducts, providing new insights into their sustainable utilization and bioactive potential.

## 1. Introduction

Polyphenols, also known as polyhydroxyphenols and a subcategory of phenolic compounds, are natural compounds and secondary metabolites that are synthesized solely by plants [1]. They generally occur as glycosidases, which are combined with divergent organic acids that help to combat pathogenic organisms [2]. They are abundant in several types of fruits, such as grapes, berries, and apples, and vegetables, such as soybeans and onions. Other than fruits and vegetables, seeds, nuts, flowers, tree barks, and several common beverages like coffee and red wine are also known for their notability for this content [3]. They have pivotal roles in many metabolic functions, such as contributing to pollinator attraction, structural functions, and several types of defense mechanisms in plants [1,4]. Their unique bioactive characteristics, including modulating oxidative stress and inflammatory response, free radical scavenging activity, and antioxidant, antimicrobial, and prebiotic properties, are widely recognized and considered significant bioactive compounds [1,5]. Polyphenols can be divided into five primary classes based on their structural characteristics and biological functions: flavonoids, tannins, stilbenes, phenolic acids, and lignans. Each category divides into several subcategories, and each polyphenol has a unique characteristic [6].

In recent years, there has been a growing number of studies, particularly in vitro assays, investigating the physiological activities of various sources of phenolic compounds, including food-derived bioactive and nutritionally functional components [3]. Furthermore, many existing techniques have been improved after their limitations, such as instability and inefficiency in freeze-drying processes, were identified [7,8]. Recently, emerging green extraction methods have been increasingly investigated to enhance phenolic compound recovery in a more energy-efficient and sustainable way. These methods aim to provide high-yield recovery, preserve thermolabile phenolics, reduce contamination, and enhance selectivity while minimizing the use of toxic organic solvents, as well as water and energy consumption. While green extraction technologies offer a more efficient and sustainable approach to recovering phenolic compounds, their effects on bioavailability depend on the extraction agents used and processing conditions. These properties make them a promising approach for pharmaceutical, cosmetic, and environmental applications, with potential applicability in food systems when food-grade extraction agents are employed. Detailed information of green methods, including their advantages and current limitations, have been discussed [9]. Although the importance of green methods has been widely recognized, only a limited number of studies were published before the 2010s, and byproduct polyphenols received little scientific attention. In recent years, however, research interest in phenolic compounds from agro-industrial byproducts has increased markedly.

To emphasize more of this content, this review article aimed to attach importance to over 100 recently published articles about the phenolic content of byproducts. To evaluate the content of the article, bioactive properties and various plant sources of polyphenols have been included. To highlight the importance of phenolic compounds from byproducts, such as widely known pomegranate, ginger, coffee, avocado, and recent findings such as *Posidonia oceanica* (seagrass) and water caltrop shells have been outlined. Their broad-spectrum sources and bioactive attributes with potential application areas such as the food and meat industry, agriculture, cosmetics, biomedicine, and health-related applications have been highlighted. Extraction techniques for phenolic compounds from plant byproducts using advanced green technologies have been compared. Limitations and challenges, with future recommendations, have also been included to facilitate the evolutionary development in the bioavailability of plant-derived polyphenols from byproducts and their application areas.

## 2. Plant Derived Phenolic Compounds and Their Bioactivities

As mentioned, polyphenols are bioactive components of versatile plant species [10]. Over the past few years, they have remarked on their various interactions, such as their binding and ridging ability or specificity, and advantageous properties, including antioxidant, anti-inflammatory, therapeutic, and antimicrobial effects [11,12]. It has also been reported that they have the ability to interfere with biochemical homeostasis and affect the epigenetic modifications of chromatin [13,14]. Stilbenes possess a 1,2-diphenylethylene backbone, which defines their chemical class, and they are known for their therapeutic properties and protective effects against plant pathogens and pests. Several examples can be given, such as resveratrol, pinosylvin, etc. [15]. Resveratrol, a non-flavonoid polyphenol, is one of the most studied stilbenoids due to the exhibition of potent antioxidant, immunomodulatory, anti-inflammatory, and antiangiogenic effects [16]. A significant number of preclinical and clinical trials have reported that it can overcome the multidrug resistance in cancer cells and has the potential to enhance the sensitivity of cancer cells to chemotherapeutic agents when it is used with other clinically used drugs [17,18]. Flavonoids, which have several subcategories named anthocyanins, flavonols, flavones, flavanones, flavan-3-ols, and isoflavones, are natural phytochemicals that are widely found in fruits and vegetables and usually identified as flower pigments [19,20]. These phytochemicals are known for their antiviral properties, and they exhibit their potent activity in various stages, including viral entrance, replication phase, or translation of proteins [21,22]. Many of these flavonoids are studied against a variety of DNA and RNA viruses due to their versatile activities [23]. It has been indicated that some of the glycoside parts of flavonoids have increased their solubility, and therefore, their antiviral activity, when they are compared to their agylcone form [24]. Furthermore, several flavonoids exhibit higher inhibitory activities without toxicity and with enhanced cell proliferation [20,25]. The divergent properties of these phytochemicals increased the amount of studies and widened as in vivo [26], in vitro [27], and in silico [28] studies. Some flavonoids, such as quercetin 3-rhamnoside (Q3R), quercetin, epigallocatechin (EGC), and baicalein, are widely used for these types of studies against various types of viruses, including influenza A and B viruses [29], Japanese encephalitis virus (JEV) [30], dengue virus (DENV) [31], hepatitis C virus (HCV) [32], and human immunodeficiency viruses (HIV) [33]. Tannins are another class of polyphenols that usually have a role in regulating plant growth and protecting the plants from predators. Condensed, hydrolyzable, and phlorotannins are three subclasses of the tannin category [34]. They have potent antioxidant, antibacterial, antiviral, and anticancer properties. Generally, tannic acid, a member of tannins, is known as a flavoring and adjuvant agent [35]. Both Gram-positive and negative bacteria have been used to investigate the activity of tannins, including (*E. coli*) *Escherichia coli*, *Staphylococcus aureus* (*S. aureus*), *Yersinia enterocolitica* (*Y. enterocolitica*), *Enterococcus faecalis* (*E. faecalis*), *Streptococcus pyogenes* (*S. pyogenes*), *Listeria innocua* (*L. innocua*), and *Pseudomonas aeruginosa* (*P. aeruginosa*) [36]. As for their antiviral properties, several trials against HIV, influenza A virus, noroviruses, papillomavirus, and herpes simplex virus types 1 and 2 (HSV-1 and HSV-2) have been investigated [37]. Furthermore, they have been used in various studies in the food industry, both in vivo [38] and in vitro [39], such as in the beef industry and as preservative agents in food [40,41], making them a potential nutraceutical. Another class of polyphenol scaffold is phenolic acids, also referred to as phenol carboxylic acids, which consist of a phenolic ring and an organic carboxylic acid function [42]. These bioactive compounds are implicated in influencing the flavor, aroma, and overall sensory profile of foods and are classified into two main subclasses: hydroxycinnamic acids and hydroxybenzoic acids [43]. Phenolic acids have gained momentum owing to their strong neuroprotective effects and potential as therapeutic agents in combating cognitive and chronic diseases, including cystic fibrosis, Alzheimer’s disease, and non-C or non-B hepatocellular carcinoma [44,45,46]. Furthermore, they exhibit antioxidant activity by preventing metal catalysis and free radical formation [47]. Finally, lignans, classified as classical lignans and neolignans [48], are commonly derived from the dimerization through oxidative reactions involving phenylpropanoid units and are known for their antiviral, anti-inflammatory, antioxidant, and anticancer activity [49]. Sesame and flax seeds consist of the highest lignan content among food groups [50]. Their anti-inflammatory activity has been tested in vitro on cyclooxygenase 1 and 2 (COX-1 and COX-2), 15-lipoxygenase (15-LOX), and phospholipases A2 (sPLA2) enzyme activities to see their inhibitory activities. Results of various assays revealed that a stronger inhibitory activity of lignans from *Schisandra rubriflora* extract exhibited anti-inflammatory activity for 15-LOX, COX-1, and COX-2 enzymes [51]. As discussed above, phenolic compounds have been investigated with various types of studies, including in vitro, in silico, and in vivo, to be able to understand the activity and expand the application areas of these bioactive compounds. One of the expanded studies has indicated that polyphenol-containing nanoparticle synthesis can be a new therapeutic approach for biomedical applications, such as in biodetection, gene delivery, bone repair, and cancer theranostics [52]. Polyphenols are highly consistent in plant-based food groups and beverages, and over 80% of polyphenol intake can be absorbed, influenced by gut microbiota. Their consumption as part of a healthy diet may help reduce the risk of chronic diseases, such as cardiovascular disease and type 2 diabetes [53]. Since they can improve endothelial function, they have also been shown to exhibit antihypertensive potential. One of the studies indicates that the improvement in endothelial function through activation of vascular eNOS and Akt signaling pathways may have an association with these effects [54]. To enhance their intake biological value and protect their structure, a research article has reviewed that the sample sizes and drying time are directly affecting their composition [55]. Moreover, according to a review article, small samples with shorter periods of drying processes conserved both the structure and nutritional value of phenolic compounds [8].

### 2.1. Antioxidant Activities of Phenolic Compounds

Phenolic compounds represent one of the major sources of daily antioxidant intake. In a healthy diet, their average consumption is approximately 1 g per day. This level is nearly ten times higher than the typical daily intake of vitamin C. Phenolic compounds can act in antioxidant action both directly and indirectly. Essentially, they can neutralize ROS directly due to their hydroxyl groups. The phenolic compound uses its hydroxyl group and donates one hydrogen atom to stabilize free radicals [56]. Phenolic compounds can also chelate metals to prevent radical formation at the source [57]. To increase antioxidant systems, several phenolic compounds have the ability to activate transcription factor Nrf2 to enhance cellular defense [58]. Moreover, enzymes that generate oxidative molecules can be inhibited by phenolic compounds to reduce cellular production of new radicals [59]. Furthermore, to protect lipid radicals and cell membrane integrity, several phenolic compounds can interrupt lipid peroxidation to prevent damage from free radicals [60]. Principal dietary contributors of polyphenols as antioxidants are fruits, vegetables, cereals, legumes, plant-based foods, and beverages, including coffee and green tea [61]. Dietary phenolic compounds have the ability to act as antioxidants due to their structure, including multiple hydroxyl groups [62]. They scavenge free radicals and reactive oxygen species (ROS) to interfere with oxidative stress, signaling pathways, and signaling transduction mechanisms of pro-inflammatory mediators [63,64]. On the other hand, some phenolic antioxidants may increase carbonyl stress and cause glycation-mediated protein damage. Therefore, the selection of specific phenolic compounds for any health-related application should be made with careful consideration [65]. In a 2012 review article, it has been pointed out that although most of the pharma and food industries have been fortifying whole foods and selling them with adequate and proven in vitro studies, more in vivo studies and clinical trials are required [66].

One of the recent research areas for the antioxidant activity of phenolic compounds derived from olives. Phenolic compounds from olives are rich in phytoalexins [67,68]. Olive oils, especially extra virgin olive oil, contain over 30 phenolic compounds, namely oleuropein, in both its glycated and aglycone (OLE) forms, verbascoside, oleocanthal, hydroxytyrosol (HT), tyrosol, etc. [69]. Recent studies indicate that bioactive components of extra virgin oil enhance cognitive function and reduce breast cancer risk. Moreover, they prevent oxidative damage, especially oxidative damage to low-density lipoproteins, by bonding with free radicals, breaking peroxidative chain reactions, enhancing cellular antioxidant defenses, and acting as metal chelators [70,71]. In addition, they also have the ability to modulate signaling pathways, including AMPK and JAG/SAT [72,73]. Several studies have also found that they are like adjuvant agents that maintain mitochondrial function and reduce inflammation, which shows that they have potential as neuroprotective agents for Alzheimer’s disease [74,75]. According to a 2023 research article, oxidative reactions that are caused by free radicals create an unpleasant odor, and synthetic antioxidants are very effective at suppressing this kind of oxidative damage by neutralizing free radicals. Nevertheless, research also indicates that more in vivo studies are needed since it can cause a harmful effect on human health [76]. In contrast, in a 2021 research article, it has been indicated that natural phenolic antioxidants usually outperform the synthetic antioxidants when it comes to food preservation from protein and lipid oxidation [77]. Due to their diverse antioxidant activity they have also been tested on animal diets. Results showed that phenolic compounds, especially flavonoids, improve animal immunity and overall gut health, contributing to healthier animal products with better fatty acid profiles [78]. Blueberry is enriched with high anthocyanins, phenolic acids, and flavonols [79]. Similar phenolic content is also found in strawberries, while it is higher in flavan-3-ols, dihydrochalcones, and flavanones [80]. When these two berries are compared, higher phenolic content has been found in blueberries with higher antioxidant activity [81]. The high antioxidant activity of blueberries has been tested using different processes. In a 2021 research article, it is proposed to investigate two different techniques of puree processing methods: high hydrostatic pressure (HHP) and thermal processing (TP). According to the results, HPP showed a better effect for maintaining the color of puree, while TP exhibited higher antioxidant activity than HPP. Furthermore, it is also indicated that a proper HHP treatment may have the ability to activate polyphenol oxidase and β-glucosidase activity [82]. To expand the phenolic content in blueberry cultivars, 14 different types of blueberry cultivars were compared by their antioxidant and phenolic content using ABTS and ferric reducing antioxidant ability (FRAP) assays [83]. According to FRAP assay results, the highest value was ≤15 mmol Trolox/100 g dw, while the highest ABTS assay result was ≤26 mmol Trolox/100 g dw. Principal component analysis results demonstrated that the Bonifacy cultivar exhibited the highest anthocyanin content, while the Aurora cultivar exhibited the highest free amino acid content. Aronia fruits, also known as chokeberries, are another berry type that is enriched with high chlorogenic acid, flavonoids (namely quercetin, catechin, and proanthocyanidins), and anthocyanin content. Generally, black chokeberry (*Aronia melanocarpa* L.) is being used to perform biological activity studies, and different parts and extract types of the fruit, including fruit, leaves, and pomace, have been used [84]. In a research article that has included different ripeness stages of aronia fruits (unripe, ripe, and overripe), the antioxidant capacity was assessed using DPPH and FRAP assays. According to results, green, unripe aronia fruits exhibited the strongest antioxidant activity, with values of 7.59 ± 0.62 in DPPH and 15.46 ± 1.34 in FRAP [85]. Similarly, blackberries are also abundant with polyphenols, namely, tannins, anthocyanins, and flavonoids. When different parts of the blackberry plant are analyzed, immature leaves of blackberry are found to have the highest phenolic content, including catechol, gallic acid, epicatechin, and rutin [86]. Varzuru et al. [87] compared the antioxidant activities of blackberry and raspberry leaves in vitro. Results demonstrated that blackberry leaves are enriched with liposoluble antioxidants, while raspberry leaves are abundant in water-soluble antioxidants, both having the capability to mitigate oxidative stress by neutralizing free radicals and inhibiting lipid peroxidation. Individually, raspberry leaves exhibited potent scavenging activity against hydroxyl radicals, while blackberry leaves exhibited high efficacy in neutralizing superoxide radicals. In another in vitro study, the objective was to determine the antioxidant activity of commercial and wild blackberries during gastrointestinal digestion [88]. Results indicated that wild blackberry digestate exhibited more than 50% antioxidant activity after gastrointestinal digestion, while commercial blackberry digestate exhibited less than 50%. Other than antioxidant activity, anticancer properties are also studied on blackberries.

Grapes are abundant with flavonoids, especially myricetin and hesperidin, and with hydroxycinnamic acid and anthocyanins, which give the darker color to the grapes. Antioxidant activity of grapes indicates that there is a strong correlation between phenolic content and fruit coloration, meaning that darker colors indicate potentially higher antioxidant activity [89]. To elaborate further, antioxidant activities of different extracts of a grape cultivar traditionally grown in Turkey ‘Karaerik,’ including peel, seed, and pulp, were investigated. DPPH analysis showed that peel extracts exhibited the highest DPPH scavenging activity, and pulp extracts had the lowest score of all three [90]. When white and red grapes were compared, different levels of bioactive compounds and bioactivities, particularly antioxidant and anti-inflammatory effects, were observed. In vitro and in vivo studies indicate that grape pomace can improve cardiac function and reduce atherosclerotic lesions by reducing ROS damage and inflammation [91]. Similarly to grapes, plums are also rich in phenolic content, with more than 20 polyphenols in both free and bound forms, including catechin, epicatechin, quercetin, neochlorogenic acid, and procyanidin B2 [92]. In a comprehensive study, the antioxidant activities of six polyphenols from the Burkedin plum, catechin, ellagic acid, gallic acid, quercetin-3-glucoside, cyanidin-3-glucoside, and vanillic acid were investigated [93]. CUPRAC (CUPric Reducing Antioxidant Capacity), TPC (total phenolic content), and FRAP assay analysis showed that ellagic acid, catechin, and gallic acid exhibited higher antioxidant activity than cyanidin-3-glucoside. Moreover, flesh extracts exhibited higher antioxidant activity than seed extracts. In a research study, apple pomace samples and their both antioxidant and prebiotic activities were investigated. According to the results, apple pomace exhibited prebiotic activity against *Lacticaseibacillus paracasei* and *Bacillus subtilis* via cell proliferation and viability. However, no significant effect on *Lactobacillus acidophilus* and *Lactobacillus plantarum* was observed. Antioxidant activity of apple pomace was tested via FRAP, ABTS, and DPPH assays. Results demonstrated a high value of trolox equivalent antioxidant capacity (TEAC), indicating that water-soluble (hydrophilic) and fat-soluble (lipophilic) antioxidants were detected in the sample. Acetylcholinesterase (AChE) is a crucial enzyme that plays a role in the nervous system by breaking down the acetylcholine (ACh) neurotransmitter [94]. This essential enzyme also remains naturally in artichokes, offering maintenance of ACh levels and antioxidant protection due to its polyphenolic content [95,96]. Yellow onion peels have antimicrobial activity against *Bacillus subtilis* (*B. subtilis*), *E. coli*, *Bacillus cereus* (*B. cereus*), *S. aureus*, *P. aeruginosa*, and *Salmonella typhimurium* with MIC values between 150 and 200 µg/mL. Moreover, when it was tested on catfish fillets, it significantly reduced lipid peroxidation and oxidative spoilage under refrigerated (4 °C) and frozen (−18 °C) storage conditions, making them a potential candidate for fish preservative application [97,98]. Lactoferrin is a multifunctional milk protein that has the ability to chelate iron, and this activity accompanies various properties, including antioxidant, antimicrobial, antiviral, prebiotic, and therapeutic effects [99]. Goat lactoferrin has been used to evaluate its effect on the solubility of phenolic antioxidants. Results demonstrated that goat lactoferrin pterostilbene complexes enhanced the solubility of pterostilbene polyphenols and became a protective layer to protect the phenolic content profile. Furthermore, improvement in the emulsification and foaming properties of goat lactoferrin has also been observed [100]. Overall, articles generally discuss the main antioxidant properties of phenolic compounds, especially radical scavenging, metal chelation, and modulation of antioxidant enzymes. Antioxidant capacity of these compounds is generally analyzed with chemical assays like DDPH, ABTS, FRAP, ORAC, which mimic in vivo or in vitro effects subtly. Moreover, it is mentioned that phenolic compounds can act as antioxidants at moderate doses and pro-oxidants at high doses. However, long-term effects of intakes are mostly poorly defined in vivo studies.

### 2.2. Antiviral Activities of Phenolic Compounds

Previous studies demonstrated that phenolic compounds exhibit immense antiviral properties due to their hydroxyl groups attached to aromatic rings, giving them the ability to scavenge ROS and chelate iron to catalyze lipid peroxidation [101]. Phenolic compounds can directly inhibit viral entry by blocking viruses from attaching or entering host cells. This mechanism can be provided by binding viral surface proteins, altering host cell membrane receptors, or preventing membrane fusion to stop the release of viral genome. EGCG binding to targeted surface protein of influenza A virus to prevent interaction of sialic acid receptor on host cells, curcumin binding envelope protein of Dengue virus to disrupt viral entry, and quercetin binding spike protein of SARS-CoV-2 to block attachment to the host cells are examples for some of these mechanisms [102,103].

Moreover, phenolic compounds such as resveratrol and quercetin, can enhance or balance immune responses by boosting interferon production like IFN-α, IFN-β, enhancing natural killer cell activity and modulating adaptive immunity to help defense mechanism [104,105]. Several phenolic compounds, especially curcumin, can interfere with host cell signaling pathways and inhibit translation factors to disrupt viral protein synthesis [106,107]. Furthermore, several phenolic compounds, such as tannins and proanthocyanidins, have the ability to inactivate viral particles and destabilize lipid envelopes of viruses [108,109]. In a recent study, over 30 polyphenols have been tested against Porcine Epidemic Diarrhea Virus (PEDV) in vitro [110]. Results indicated that four of them exhibited anti-PENDV activity and have the potential to be candidates for anti-PENDV drugs. Phenolic compounds, including resveratrol, cyanidin, ellagic acid, and luteolin, can act as inhibitors against hepatitis B, influenza, HCV, HIV, severe acute respiratory syndrome coronavirus 2 (SARS-CoV-2), DENV, zika virus (ZIKV), and chikungunya virus by hindering viral growth and replication [101,111,112]. In a recent study, over a hundred phenolic acids, with nearly sixty abundant polyphenols, were investigated in nearly twenty Indian heritage pigmented rice varieties (IHPRV) via LC-Q-TOF-MS (a high-resolution metabolomic profiling technique) to examine their antiviral activity against SARS-CoV-2 [113]. Results indicated that nearly twenty of the abundant polyphenols exhibited antiviral activity. To date, many phenolic compounds have been studied for their diverse antiviral activity. One such compound that has been studied for its various benefits, including antiviral activity, is epigallocatechin gallate (EGCG), which is abundantly found in green tea [114]. It has been indicated that EGCG has a potent activity against DENV, HIV, chikungunya virus, SARS-CoV-2, and ZIKV viruses [115]. To elaborate, in silico studies reported that EGCG can inhibit ZIKV growth by suppressing viral entry. Further study by Kumar et al. indicated that EGCG can also interrupt various stages of replication in certain viruses, giving an exemplification of EGCG activity against ZIKV on NTPase enzyme inhibition with an IC50 value (half of the maximal inhibitory concentration) of 295.7 nM and a Ki value (binding affinity of an inhibitor to its target receptor or enzyme) of 0.387 ± 0.034 μM [116]. A recent study aimed to investigate EGCG activity against Mayaro virus, using BHK-21 and Vero E6 cell lines in vitro and in silico [117]. Results demonstrated that EGCG suppressed the Mayaro virus replication and exhibited antiviral activity in the entry and post-entry stages. Similar studies include the antiviral activity of curcumin against HCV via preventing virulence attachment [118], human herpesviruses (HHV) by hindering replication or blocking viral entry [119], and Nipah virus by hindering RNA synthesis [120], and aronia extracts against influenza virus types (type 1 and 3) by inhibiting surface proteins of the virus [121].

In a recent in vitro study, several plant extracts, essential oils, and their phenolic compounds, especially quercetin and coumarins, have been investigated for their antiviral activity against certain virus types [122]. According to an investigation, quercetin can inhibit replication of several virus types, especially influenza virus types, HSV, SARS-CoV-2, porcine viruses, and HCV [120,123], while coumarins show potent activity against HIV-1 (HIV type 1) [124]. Moreover, essential oils demonstrated immense activity against different virus types, such as tea tree oil against HSV, influenza, and human papillomavirus (HPV) [125]; eucalyptus oil against HSV, influenza, and respiratory syncytial virus (RSV) [126]; peppermint oil against HSV, influenza, and adenovirus [127]; lavender oil against HSV and influenza; oregano oil against HSV and norovirus [128]; and lemon balm oil against enterovirus and HSV [122,129]. It has also been indicated that studies of essential oils exhibited different types of antiviral activity, including direct inhibition of viral growth, suppressing viral replication, inactivation of viruses, and inhibition of viral attachment [122]. Kreiser et al. investigated a group of structurally related phenolic compounds, including EGCG, quercetin, taxifolin, and naringenin. These compounds were evaluated in combination with zinc picolinate and copper sulfate. The study aimed to assess their antiviral efficacy against respiratory RNA viruses, specifically influenza A, human metapneumovirus, and human coronavirus OC43 [130]. These combinations were tested on two groups, one with naringenin and another without naringenin. Results demonstrated that the combination with naringenin reduced viral replication of human metapneumovirus, human coronavirus OC43, and influenza A virus via potentially acting as ionophores to enhance intracellular zinc levels. *Morus alba*, known as white mulberry, consists of highly flavonoids such as quercetin, kaempferol, and catechins, and phenolic acids like caffeic acid and chlorogenic acid. The antiviral activity of white mulberry was tested against pseudorabies virus (PRV), and significant inhibition of viral replication was observed [131]. The polyphenols in turmeric and red grapes, such as catechin, resveratrol, curcumin, and eriodictyol, have a strong affinity for ACE2 ligands [121,132]. These polyphenols block the replication cycle by suppressing the activity of the protease to hinder the ssRNA replication. Additionally, polyphenols, especially quercetagetin, myricetin, and EGCG, exhibit high affinity for SARS-CoV-2 RdRp (RNA polymerase of SARS-CoV-2) [133,134].

Polyphenol-rich sugarcane extract (PRSE) is another compound that is widely studied for antioxidant, antimicrobial, and antiviral activity research due to its high phenolic content. Tang et al. studied PRSE against influenza A to investigate its antiviral activity in vitro [135]. For this purpose, A549 (human lung epithelial) and MDCK (Madin-Darby Canine Kidney) cell lines were used against H3N2 and H1N1 strains. Results showed that PRSE acts as an inhibitor in the early stages of the viral replication cycle and can suppress the replication of both strains. To expand the knowledge, a year after this research, Tang et al. also studied PRSE on respiratory virus types, namely RSV, Parainfluenza Virus Type 3 (PIV-3), Adenovirus 5 (AdV5), and SARS-CoV-2 [136]. In this study, the A549, Calu-3, Vero E6 (for SARS-CoV-2), and HEp-2 (for RSV and PIV-3) cell lines were used. Results showed that the strongest inhibition was determined against SARS-CoV-2 and influenza A virus by affecting the early stages of viral replication. In contrast, little to no inhibitory effect was determined against RSV, PIV-3, and AdV5. Monkeypox virus (MPXV) is a zoonotic disease that is predicted to cause a potential global pandemic in the near future [137]. The F13L gene of MPXV encodes an envelope protein named F13, which is crucial for its viral replication cycle, and since phenolic compounds exhibit immense antiviral activities, an in silico study investigated over five polyphenols and their activity against F13 [138]. According to molecular docking results, myricetin, demethoxycurcumin, piceatannol, curcumin, matairesinol, and ellagic acid exhibited strong binding activity to F13, and more stable complexes were observed with demethoxycurcumin and myricetin, making them powerful candidates for inhibiting the F13 protein of MPVX. Due to their diverse antiviral activities, Manzoor et al. [139] investigated several compounds, including flavanols, flavonoids, and coumarins, against various fish viruses, namely viral hemorrhagic septicemia virus (VHSV), infectious hematopoietic necrosis virus (IHNV), grass carp reovirus (GCRV), singapore grouper iridovirus (SGIV), infectious pancreatic necrosis virus (IPNV), and spring viremia of carp virus (SVCV). Results showed that EGCG blocks the adhesion entry of GCRV, quercetin exhibited potent antiviral activity against SGIV and IPNV, and flavonoids such as fisetin, fustin, and sulfuretin were able to induce apoptosis of the infected cells of IHNV and VHSV. Even though the studies have expanded their knowledge and findings by demonstrating antiviral activities against several viruses and being potential antiviral candidates, further research is needed on the variability in dosage, effectiveness, and safety of phenolic compounds [140]. Recent antiviral articles highlight mechanism clearly and discuss strong in vitro signals. Nevertheless, comparative mechanism work between enveloped and non-enveloped viruses, RNA and DNA viruses are only just emerging without clear knowledge about action of phenolic compounds, whether viral-specific targets or not.

### 2.3. Antimicrobial Activities of Phenolic Compounds

Antimicrobial agents and therapies are globally crucial to overcome health challenges. To date, phytochemicals are widely studied for this purpose due to their ability to disrupt bacterial cell membranes, generate ROS, interfere with RNA synthesis, and block signaling pathways [141]. Phenolic compounds act against bacteria through multiple direct and indirect mechanisms involving cell wall, membrane, enzymes, metabolism, and pathways. They can damage structural component of bacteria and cause rapid cell lysis by disrupting cell permeability, increasing membrane rigidity, causing leakage of ions or metabolites, and damaging peptidoglycan layers [142,143]. By inhibiting essential enzymes like DNA gyrase or ATP synthase, they can cause impaired bacterial growth and inhibition of replication [144]. Other than enzymes, phenolic compounds can also directly bind bacterial proteins for denaturation, formation of irreversible complexes, and alteration in functional domains [145]. Moreover, several phenolic compounds can interfere with metabolic pathways to reduce bacterial viability by disrupting electron transportation, reducing ATP generation, and inhibiting fatty acid synthesis [146]. Furthermore, phenolic compounds can inhibit the biofilm that protects bacteria against antibiotics and immune defenses [147]. In addition to antioxidant activities of blueberry cultivars, their antimicrobial activities against Gram-positive bacteria, including *Bifidobacterium longum* (*B. longum*), *Faecalibacterium prausnitzii*, *Enterococcus hirae*, *E. faecalis*, *S. aureus*, *B. subtilis*, and *B. cereus*, and Gram-negative bacteria, including *P. aeruginosa*, *Vibrio harveyi*, *Salmonella enterica* subsp. (*Salmonella* ssp.), and *E. coli* ATCC 10536, were tested. Results showed that the Aurora and Nelson cultivars exhibited the highest antimicrobial activity for *B. cereus*, *P. aeruginosa*, and *Vibrio harveyi*, while the Duke and Bonus cultivars showed no antimicrobial activity. Similarly, another study proposed to investigate two different berry extracts, *Prunus spinosa* L. and *Vaccinium corymbosum*, and their antimicrobial activities via performing the minimum inhibitory concentration (MIC) method on *Shigella flexneri* ATCC 10708, *Salmonella choleraesuis* ATCC 12022, and *E. coli* ATCC 12792 [148]. According to results, *Prunus spinosa* L. extracts showed the same MIC of 250 µg/mL for *E. coli* ATCC 25922 and *Shigella sonnei* ATCC 25931, while *Vaccinium corymbosum* extracts showed different MIC results for *Salmonella enteritidis* ATCC 13076 with 2.16 mg/mL and *E. coli* ATCC 25922 with 3.74 mg/mL. Similarly, apples are also one of the studied fruits in this aspect, due to their myricetin, catechin, t-ferrulic acid, cinnamic acid, etc. [149]. When it is studied in different formulations, namely three different extract forms—hydroethanolic, microwave-assisted, and purified polyphenolic extracts—different MIC values were observed against three pathogens [150]. Results showed that apple pomace exhibited antimicrobial efficiency against *S. aureus* with an MIC value of 2.5 mg/mL and *Shigella flexneri* (*S. flexneri*) with an MIC value of 0.625 mg/mL. Norway spruce bark is a pyramidal tree that has weeping branches. Recent studies highly investigated these trees due to their phenolic content, including stilbenes, tannins, resin acids, etc., and bioactivity features. A recent study examined two methods to compare their extraction efficiency: hot-water extraction (HW) and hydrodynamic cavitation (HC). Results showed that the HW method yielded higher total dissolved solids when it was compared to the HC method. Antimicrobial activity was tested against one Gram-positive, *S. aureus*, and one Gram-negative bacterium, *E. coli*. Antimicrobial activity tests indicated that HC extracts showed higher activity against HW extracts [151]. Pomegranate is another polyphenol-rich fruit that has high punicalagin, ellagic acid, and gallic acid [152]. Biological activities of pomegranate use various extraction types, including peel, flower, juice, pomace, etc. Predominantly, 18 polyphenols and 11 flavonoids have been identified in pomegranate peels [153,154]. This high polyphenolic content accompanies antioxidant, antibacterial, and anti-inflammatory properties [155,156]. According to studies, tannins play a crucial role in antimicrobial and anti-inflammatory activities, especially antimicrobial activity against methicillin-resistant *S. aureus* (*MRSA*) [157]. Additionally, its antioxidant activity helps to mitigate inflammation, making it a powerful candidate for antibiotics in managing respiratory infections [158]. In an in vivo study, Yassin et al. reported that pomegranate peels exhibit antimicrobial properties against *S. aureus* with 0.125 mg/mL and *MRSA* with a 0.250 mg/mL minimum inhibitory concentration (MIC) value. Besides the peel extracts, the antioxidant capacity and antimicrobial properties of pomegranate flower extracts were also investigated. Zhang et al. investigated the antimicrobial activity of pomegranate flowers against *Streptococcus* mutans. Results indicated that flower extracts inhibited biofilm formation, and as the concentration increased, inhibition zones grew larger [159].

Rosemary hydroalcoholic macerates were studied for the purpose of comparison between creams and hydrogels and evaluation of their antimicrobial, anti-inflammatory, and antioxidant properties [160]. Two types of rosemary, from the Romanian and Bulgarian coastal regions, were tested in different ethanol concentrations, which were supplied to also compare geographic origin. Results showed that rosemary, especially the Romanian species with 70% ethanol extracts, showed potential to evaluate bioactivity properties with high polyphenol content, namely, phenolic acids and flavonoids, which significantly exhibited antimicrobial activity against *Candida albicans* (*C. albicans*), *S. aureus*, and *E. coli*. In contrast, Bulgarian extracts showed no significant antimicrobial activity, indicating that geographic regions are also important for their phenolic content and bioactivities. As mentioned previously, catechins are widely studied and involved in various plant sources, namely catechin, epicatechin (EC), EGC, EGCG, epicatechin gallate (ECG), etc. [161]. They are considered antimicrobial agents due to their diverse action against different pathogens, including disrupting cell walls and membranes of the bacteria, hindering viral expression, suppressing DNA damage, ROS mechanisms, and their synergistic activities with antibiotics [162]. It has been demonstrated that their MIC values are around 1–2 mg/mL, and their minimum bactericidal concentration (MBC) values are around 2–4 mg/mL, depending on the bacteria. When it is compared to MIC, it shows the lowest concentration of an antimicrobial agent that inhibits visible bacterial growth in a subcultured antibiotic-free media [163]. Furthermore, the combination of catechins with tetracycline, erythromycin, clindamycin, azithromycin, vancomycin, and gentamicin exhibited synergistic effects with catechins [164]. To elaborate further, catechins have the ability to produce hydrogen peroxide, which is a compound that causes oxidative damage to the cells and causes an indirect kill. However, if a bacterium has a catalase enzyme, it will break down hydrogen peroxide into water and oxygen to evaluate the survival of bacteria [165,166]. As for hindering biofilm formation, catechin presence blocks the acrA gene, which is resistant to many drugs and causes the formation of biofilm in *E. coli* [164,167]. In a recent study, the polyphenolic content of *Osmanthus fragrans* was analyzed [168]. Results reported that two types of phenolic compounds were found in major quantities: phenolic acids that exhibit both antimicrobial and ROS-generating activity, and flavonoids that exhibit antifungal activity. Further study is needed to investigate its potential in food packaging by studying its activity against a fungal pathogen named *Alternaria alternata*. The results indicated that phenolic compounds from *Osmanthus fragrans* blocked spore germination, making it a candidate as an antimicrobial agent in food packaging. In a comparative review article about in vivo and in vitro studies, flavonoids, lignans, stilbenes, and phenolic acids were involved. Due to their unique structures and binding conditions, it has been demonstrated that they exhibit antimicrobial activity against not only *S. aureus* but also other foodborne Gram-positive pathogens, including *Clostridium botulinum* (*C. botulinum*), *Clostridium perfringens* (*C. perfringens*), and *Listeria monocytogenes* (*L. monocytogenes*). Other than Gram-positive, they also show potent activity against several Gram-negative bacteria, such as *Campylobacter* spp., *Salmonella* spp., *Vibrio* spp., and *E. coli.* Since they are widely studied in both in vitro and in vivo studies in a dose-dependent manner, the European Food Safety Authority (EFSA) has started to analyze their potential as antimicrobial resistance activity by investigating them on various bacterial strains and conditions [169,170]. Lund et al. studied the glycosylation process and its effect on the antimicrobial activity of quercetin. Results of the study demonstrated that it significantly affects the antimicrobial activity of quercetin, and when it is compared with glycosylated quercetin, non-glycosylated quercetin exhibited stronger antimicrobial activity against *E. coli* [171]. Other than plant sources, marine sponge species *Dysidea etheria* (*D. etheria*), *Echinodictyum dendroides* (*E. dendroides*), and *Desmapsamma anchorata* (*D. anchorata*) are also studied for their potential polyphenol content. Unexpectedly, extracts contained a low amount of polyphenol content. When their antimicrobial activity was tested, an MIC value of nearly 1024 μg/mL was obtained, indicating weak direct antimicrobial activity against the tested pathogens, and they exhibited significant activity against the *multi-resistant bacterium* *Pseudomonas aeruginosa* [172]. To overcome biomedical challenges like low bioavailability of bioactive compounds, microbial infection risks, and poor mechanical strength, multifunctional hydrogel systems were aimed to be developed [173]. For this purpose, Germi et al. used polyphenolic nanosheets that are derived from propolis and placed them into a gelatin methacrylate (GelMA) matrix. High polyphenolic content from flavonoid and phenolic acid levels from propolis helped the gel to involve characteristics like antimicrobial, antioxidant, and anti-inflammatory activity. To enhance their antimicrobial activity via cationic interactions, bis-imidazolium ionic liquid (BIm-IL), and to evaluate mechanical strength in hydrogel, 3-methacryloxypropyltrimethoxysilane (MPS) was used. These functionalities developed this hydrogel as a multifunctional system, making it a strong candidate for biomedical applications. To elaborate further, a recent review article compared both animal- and plant-derived polyphenol-rich extracts and their diverse effects on gel systems, including hydrogels, oleogels, nanogels, films, and hybrid gels [174]. According to the studies, combinations of gels and bioactive compounds from diverse developments include higher antioxidant activity with higher 3-ethylbenzothiazoline-6-sulfonic acid (ABTS) and 2,2-diphenyl-1-picrylhydrazyl (DPPH) scavenging activity and antimicrobial activity against *S. aureus*, *E. coli*, *C. albicans*, etc. Even though their combination opened a new perspective on gel systems, it has been crucially indicated that variability, stability, release control, and bioavailability challenges, as well as interaction or scale-up problems, are still yet to be determined. Generally, the literature identifies clearly that several phenolic compounds can disrupt membranes, chelate metals, and inhibit enzymes. However, specific binding sites or molecular targets and structure–activity relationships are generally poorly defined. Each study used appropriate extraction methods, solvents, organism strains, and MIC protocols, depending on their purpose. Although this gives variability to literature, it makes direct comparison, generation of high variable MIC values difficult. Moreover, in real foods, wound dressings, or formulations, phenolic compounds interact with proteins, lipids, and polysaccharides. These interactions can reduce free active concentration or change how they reach microbial cells, but most mechanistic work is performed in simple buffer systems, presenting a huge knowledge gap for application areas.

### 2.4. Therapuetic Potential of Phenolic Compounds

Antimicrobial, antiviral, anti-inflammatory, and antioxidant properties of phenolic compounds led them to be studied in diverse fields and made them study in various global health challenges, as well as the food industry, including food preservation and packaging. In a review article, it has been summarized that EGCG reduces ROS damage, curcumin supports synaptic function, resveratrol acts as a protectant for mitochondrial function, and anthocyanidins support synaptic plasticity, showing a potential for neurodegenerative disease treatments, including Parkinson’s disease, Alzheimer’s disease, Huntington’s disease, and multiple sclerosis [175]. In another review article, it has been mentioned that several phenolic compounds, including resveratrol, piceatannol, oleuropein, ferulic acid, propolis, green tea polyphenols, hesperidin, etc., can act as neuro-, photo-, and gastroprotectants with significant anticancer activities [176]. However, poor bioavailability and clinical efficiency need further experiments to evaluate the delivery efficiency, including nano-formulations, microencapsulation, and dietary strategies. Moreover, it has been demonstrated with various studies that phenolic compounds prevent cardiovascular diseases, neurological diseases, liver diseases, and diabetes [177,178]. To enhance their bioavailability and nutritional value, bio-based polymers like proteins and polysaccharides are used for nanocarriers [179]. A life-threatening inflammatory disorder, acute pancreatitis, has been studied with phenolic compounds in preclinical trials [180]. According to results, it has been demonstrated that they have the ability to manage acute pancreatitis, but due to a lack of clinical trials, bioavailability, dose dependency, and therapeutic efficiency for acute pancreatitis are still unexplored. Fortunately, Dong et al. studied the formation of a polyphenol-rich nanocarrier to enhance the effectiveness of gemcitabine (Gem) and the bioavailability of polyphenol in pancreatic cancer [181]. For this study, a natural polyphenol, 1,2,3,4,6-pentagalloyl glucose (PGG) is used. For in vivo studies, mouse and PDX models (patient-derived xenografts) were utilized, whereas PANC-1 and Panc02 cancer cells were used for in vitro studies. According to results, the combination of this PGG nanocarrier increased the bioavailability and stability of PGG while increasing apoptosis (a regulated form of cell death) and inducing immunogenic cell death. Plant-derived vesicle-like nanoparticles are also studied for determining their diverse anticancer activities [182,183]. Accordingly, phenolic compounds from tea flowers or bitter lemon exhibit anticancer activity by causing apoptosis via ROS damage or cycle arrest and supporting the immune system [184]. Moreover, phenolic compounds from cannabis with high cannabidiol (a chemotype) content demonstrated a significant preventative effect against hepatocellular carcinoma, which is a type of liver cancer [185]. To effectively examine the bioavailability, functionality, and stability of phenolic compounds for acne therapy, they have been encapsulated into nanocarriers [186]. According to results, polyphenols, including flavonoids, showed significant anti-inflammatory activity by inhibiting COX-2, interleukin-6 (IL-6), nuclear factor kappa B (NF-κB), interleukin-1 beta (IL-1β), tumor necrosis factor-alpha (TNF-α), and prostaglandin E2 (PGE2) pathways; antibacterial activity by targeting *Propionibacterium acnes*; and antioxidant activity by scavenging ROS. Furthermore, the prevention of sebum oxidation was also observed. Phenolic compounds were also studied in delivery methods, including hydrogels, scaffolds, nanoparticles, and nanofibers, to compare their efficiency for acute and chronic wound healing [187]. Hydrogels induced a sustained drug release, while scaffolds supported cell proliferation. Moreover, nanoparticles protected the unstable polyphenols from unstable forms, while nanofibers were able to mimic the extracellular matrix structure, becoming more prevalent in diabetic wounds. Froldi et al. studied several plant-derived phenolic compounds, namely curcumin, baicalein, mangiferin, resveratrol, and quercetin, to investigate their efficiency for the prevention and treatment of a vascular disease, peripheral artery disease [188]. Results showed that baicalein and quercetin reduced inflammation by inhibiting NF-κB and TLR4 signaling pathways, as well as oxidative stress with nearly no toxicity, while curcumin was reported with liver toxicity in high doses. Mangiferin was also able to reduce ROS damage and inflammation by inhibiting the same signaling pathways dose-dependently, with several skin side effects from the mango extract. Resveratrol exhibited strong endothelial, anti-inflammatory, and antioxidant effects, but it has been reported that high doses (more than 150 mg/day) may cause thyroid issues. Bioactive compounds, namely flavonoids, alkaloids, and terpenes, exhibit unique multi-target activity by reducing neuroinflammation, improving neuroplasticity, and enhancing mitochondrial function and cognition. These properties make them a promising candidate for potential in Alzheimer’s disease prevention and therapy. However, it is noted that, similarly to other clinical trials with phenolic compounds, bioavailability, stability, and delivery challenges still remain [189]. To study mitochondrial function and its effect on barrier integrity, polyphenols of kiwifruit were tested with lipopolysaccharide (LPS) in both in vivo studies that included LPS-stressed rat models and in vitro studies that included Caco-2 cell lines [190]. According to the results, it has been demonstrated that kiwifruit polyphenols improve mitochondrial function and inhibit mitophagy that is driven by AMPK/ULK2, making them a candidate food natural nutraceutical as a gut barrier protectant and for intestinal disorders that are linked to mitochondrial function. In a recent study, the pro-apoptotic activity of a blackberry species (*Rubus adenotrichos*) in liver (HepG2), stomach (AGS), skin (SK-Mel-28), and colon (SW-620) cancer cell lines were investigated. Results demonstrated that fruit and pulp extracts had strong cytotoxic effects, with IC_50_ < 200 μg/mL [191]. In another recent in vitro study, anti-inflammatory, antioxidant, and autophagy-modulating effects of Andean blackberry polyphenols were proposed [192]. In this study, a dose of 80 μg/mL Andean blackberry extract was used, and the results demonstrated an inhibition of the NLRP3 inflammasome, with reduced inflammation and oxidative stress. Hypoglycemic polyphenols that have been indicated as having an essential role in the inhibition of carbohydrate digestion and insulin stimulation are abundant in walnuts [193,194]. Hu et al. analyzed these hypoglycemic polyphenols derived from walnut green husk. Results reported that they enhance glucose uptake and glycogen accumulation in insulin-resistant HepG2 and 3T3-L1 cells. Moreover, their inhibition of α-glucosidase and α-amylase has also been observed [193,195]. As previously mentioned, pomegranates are rich in phenolic content and its anticancer properties were reported by exhibiting antiproliferative activity against MCF7 breast cancer with an IC50 value of 8.15 µg/m. [196]. In addition to these studies, various types of nanoparticles, especially silver nanoparticles, have been used with extracts like pomegranate peel to develop potential antimicrobial agents and antioxidants. These trials are also crucial for being green synthesis studies, meaning eco-friendly methods are being used in this research [197]. Onion is also rich in quercetin and other phenolic acids, giving antifungal and antioxidant activities, particularly against *Ascosphaera apis*, a bee pathogen [198]. A comparison between five different onion cultivars (Tank, Cobra, Bomul, Hongbanjang, and Gujji) and their different parts (root, peel, and bulb) showed that peel had the highest phenolic content among all cultivars with abundant quercetin, caffeic acid, and protocatechuic acid content [199]. Bozinou et al. [200] investigated onion skins to enhance recognition of their therapeutic potential by studying their antioxidant, antimicrobial, anti-inflammatory, and anticancer properties. In vitro studies on U-87 MG (human glioblastoma) and MCF-7 (human breast cancer) demonstrated that extracts with more than 25 µg/mL concentrations significantly suppressed cancer cell viability. Moreover, all tested onion skin samples exhibited dose-dependent cytotoxicity, making them potential adjuvants for cancer therapy and nutraceutical development [200]. Green tea is a widely studied plant for its diverse properties due to its various contents, including phenolic compounds [201]. In a study where it was compared with black tea, green tea showed the highest antioxidant activity due to its higher amount of EGCG, EC, EGC, and catechin content. Moreover, it is indicated that EGCG in green tea corresponds with apoptosis via p53 and caspase pathways. Additionally, when it is tested on MDA-MB-231 breast cancer cells via MTT (3-(4,5-dimethylthiazolyl-2)-2,5-diphenyltetrazolium bromide) assay, green tea caused high cell death at 100–200 μg/mL, compared to black tea samples [202,203]. Nevertheless, it is notable to say that synergy, additive effects, or interference with drug metabolism of polyphenol interaction against standard antiviral drugs are limited for therapeutical applications.

## 3. Application Areas of Phenolic Compounds from Plant Byproducts

Until recently, phenolic compounds from byproducts were associated with low production efficiency, even though these bioactive compounds show immense potential for practical applications. Industries increasingly face pressure to minimize waste, create circular economic systems, and use byproducts, especially from peels, seeds, pomace, husks, into value-added ingredients for food, pharmaceuticals, and cosmetics (Table 1).

### 3.1. Extraction Methods

#### 3.1.1. Conventional Extraction Technologies

Traditional extraction methods such as maceration, Soxhlet extraction, and reflux extraction have been widely applied due to their simplicity and low equipment requirements. However, their long extraction times and extensive solvent consumption limit their efficiency and sustainability compared to emerging green technologies. In addition, they rely on large amounts of toxic organic solvents, high energy use, and harsh processing conditions that can damage sensitive bioactive compounds and generate environmental burdens.

#### 3.1.2. Green Extraction Technologies

**Figure 1:**
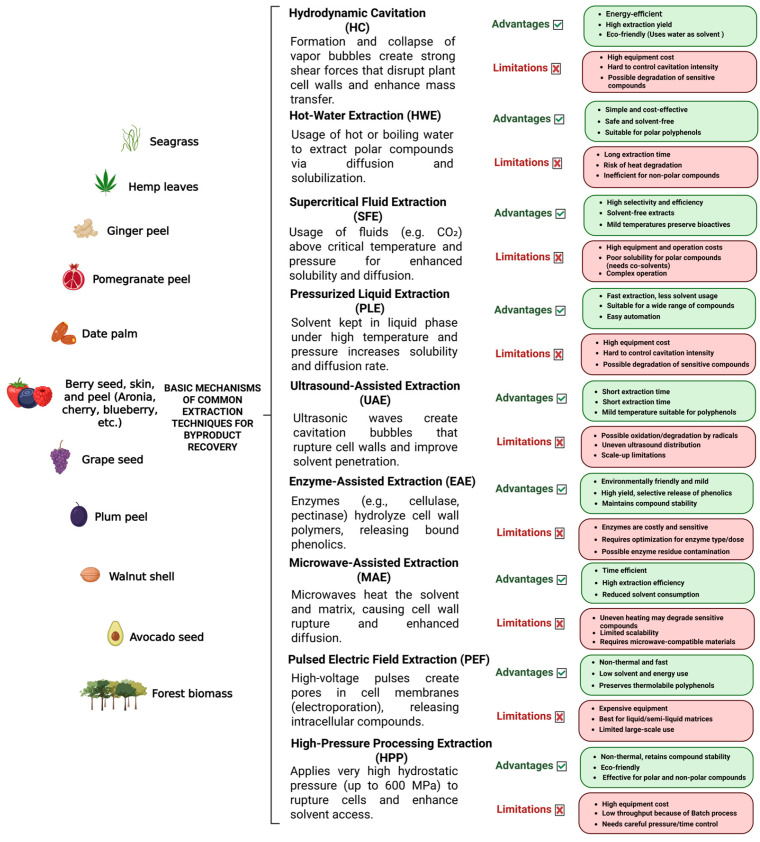
A comparative overview of different extraction techniques for polyphenol recovery, highlighting their main principles, advantages, and limitations.

Green extraction methods use safer solvents, lower temperatures, and reduced energy inputs, allowing higher recovery of stable, high-quality phenolic compounds. Green extraction methods provide this valorization by making the process cleaner, scalable, compliant with regulatory expectations, and more aligned with consumer demand for natural, sustainable products (Figure 1) [9,242,243]. Upon analysis of plant materials, hydrolytic treatment gives an easier polyphenol recovery of non-extractable phenolic compounds. Bound phenolic compounds such as proanthocyanidins and phenolic acids like ferulic acids, are chemically attached to cell wall components in plant tissues that cannot be extracted easily without hydrolysis. Once released, they exhibit higher bioactivities compared to free ones [244].

High-Pressure-Assisted Extraction (HPAE), a related technique, offers an eco-friendly alternative to conventional heat treatments by reducing extraction time, solvent use, and energy consumption while increasing yield [245]. In comprehensive research, optimum conditions of HPAE for phenolic compounds from olive pomace has been investigated [246]. Results showed that HPAE emerged as a more efficient and environmentally friendly extraction method than traditional solvent extraction for recovering phenolic compounds from olive pomace. Overall, HPAE method enhanced the yield and antioxidant activity of phenolic compounds from olive pomace without altering chemical structures, making it a promising green technology for valorizing olive industry byproducts.

HC is a green and energy-efficient technology that uses controlled formation, growth, and collapse of vapor bubbles in a liquid flow to enhance physical and chemical processes. It has been used particularly for extraction, mixing, cell disruption, and biomass valorization [247]. An extensive review article aimed to summarize and evaluate HC for extracting and valorizing valuable compounds from plants and biomass [248]. The summary suggests that HC significantly enhances mass transfer, cell wall rupture, and matrix disintegration, resulting in higher extraction yields of bioactive compounds compared to conventional or ultrasonic extraction. Moreover, it demands less energy than UAE and can be easily scaled up for industrial-level operation with simple configurations and reduced solvent use. It represents a promising tool for green chemistry, biofuel production, and circular bioeconomy applications, as well as a powerful technique for eco-friendly valorization of plant and biomass materials. Nevertheless, current limitations include equipment erosion, process optimization complexity, variability among feedstocks, and limited industrial-scale data. Overcoming these requires better reactor design, process modeling, and integration with complementary green technologies.

Supercritical Fluid extraction (SFE) is a green extraction technique that utilizes fluids in conditions above their critical temperature and pressure, where they exhibit both gas-like diffusivity and liquid-like solvating power. SFE enables efficient extraction under mild conditions, minimizing the degradation of heat-sensitive phenolic compounds. The process occurs in a closed, oxygen-free system, reducing oxidation and light-induced degradation. Additionally, solvent recovery is simple, as CO_2_ reverts to gas upon depressurization, allowing for reuse and reduced solvent waste. Because CO_2_ is a non-polar solvent, its ability to extract polar compounds such as polyphenols is limited. To overcome this, small amounts of polar co-solvents, especially ethanol, are often added, which slightly increases complexity and cost [249,250]. In a comprehensive study, recovery of tannins from various biomass resources using SPE was investigated. Results highlighted that SFE enables highly selective extraction of specific tannin fractions (hydrolyzable vs. condensed tannins) by adjusting pressure, temperature, and co-solvent composition. When reactivity and byproducts are analyzed, it has been suggested that under supercritical or subcritical conditions solvent reactivity (especially water) can cause secondary reactions such as hydrolysis and depolymerization, generating gallic acid, ellagic acid, and sugars from complex tannins. These reactions can either lower yield or create valuable derivatives, depending on the application [251].

Pressurized Liquid extraction (PLE), also known as Accelerated Solvent Extraction (ASE), is an automated and environmentally friendly extraction technique that employs high pressure and temperature to enhance the efficiency of compound recovery. Elevated pressures allow solvents to remain in the liquid phase above their boiling points, which increases solubility and diffusion rates of target compounds while reducing solvent viscosity and surface tension, thereby facilitating efficient extraction from the matrix. PLE is recognized as a rapid, economical, and efficient alternative to conventional extraction methods, often yielding equal or higher concentrations of phenolic compounds. The method can be operated in dynamic or static modes, providing flexibility and reproducibility. Despite its efficiency, the high temperature and pressure conditions may risk degradation of thermolabile compounds, and the requirement for specialized equipment increases initial operational costs [252]. A large-scale study was conducted to develop and optimize a sustainable PRE process using ethanol–water mixtures as green solvents for recovering bioavailable phenolic antioxidants from grape seed byproducts [253]. Evaluation of the bioavailability and stability of phenolic compounds during in vitro digestion and Caco-2 cell transport, linking extraction conditions with biological relevance, was also investigated. Results highlighted that optimized low-temperature PLE (75% ethanol, 20 °C, 11 min) efficiently extracts bioavailable antioxidant phenolics from grape seed byproducts with minimal degradation and strong sustainability advantages. This process supports circular economy valorization of winery waste and offers a scalable green-technology route for functional-ingredient production. Nevertheless, to achieve industrial viability and biological relevance, future research must focus on optimizing bioavailability, enhancing energy and solvent efficiency, and integrating PLE into holistic circular bioeconomy frameworks.

Ultrasound-assisted extraction (UAE) is a modern extraction technique that uses acoustic cavitation, which refers to the formation and collapse of microbubbles caused by ultrasonic waves, to disrupt plant cell walls and enhance the release of intracellular compounds. UAE enables efficient recovery of compounds bound within the plant matrix and prevents thermal degradation of bioactive molecules due to its operation at relatively low temperatures. The efficiency depends on ultrasonic parameters (frequency, intensity, and duration), and overexposure can lead to partial degradation or oxidation of sensitive compounds. Additionally, scalability for industrial applications remains a technical challenge [254]. In a research article where two potato peel varieties (Lady Claire and Lady Rosetta) are used, UAE with traditional solid–liquid extraction (SLE) tested different ultrasonic frequencies (33 and 42 kHz) have been investigated [255]. Results showed that UAE significantly improved phenolic recovery and antioxidant activity compared to SLE. Moreover, research proved that UAE is a faster, more effective, and environmentally friendly method under 33 kHz than 42 kHz for obtaining antioxidant-rich phenolic compounds from potato processing waste.

Microwave-Assisted Extraction (MAE) has emerged as a highly efficient technique for isolating bioactive compounds from food matrices. This method offers significant advantages over conventional extraction processes, including reduced extraction time, lower solvent consumption, and enhanced automation. The integration of ultrasonication with MAE further improves extraction efficiency by increasing yield and minimizing component degradation. Modified MAE systems address the limitations of traditional approaches, offering solvent-free or low-solvent operation, higher extraction efficiency, and greater energy savings. Overall, MAE provides a rapid, uniform, and energy-efficient means of extracting bioactive compounds under optimized conditions [256,257]. A research article aimed to optimize UAE of phenolic compounds from Annona muricata (soursop) leaves to evaluate their antioxidant and antimicrobial properties [258]. The parameters that are included in this study were extraction time, temperature, and solvent concentration to optimize the conditions and investigate their influence on yield of bioactive compounds. Results showed that UAE exhibits as a rapid, sustainable, and effective method for extracting antioxidant and antimicrobial compounds from Annona muricata leaves.

Enzyme-Assisted Extraction (EAE) utilizes the catalytic activity of specific enzymes to hydrolyze the components of the cellular matrix, thereby breaking down cell walls and facilitating the release of intracellular metabolites into the surrounding medium. The process generally involves the use of a solvent, which may be either organic or aqueous in nature, to enhance the penetration and recovery of target compounds. EAE offers several advantages, including effective cell wall disruption, selective extraction of metabolites, mild operating conditions, and improved extraction yield and rate. However, despite these benefits, large-scale application of EAE remains limited due to several drawbacks. These include the high cost of enzymes, incomplete cell wall degradation that necessitates additional purification steps, enzyme instability under certain processing conditions, limited recyclability and reusability, and loss of catalytic activity after repeated use [259]. To improve the release of insoluble-bound phenolic compounds from winemaking byproducts (grape skins and seeds), a study investigated the influence of EAE method [260]. To determine their efficiency in improving phenolic yield, antioxidant activity, and enzyme inhibition, two enzymes Pronase (a protease) and Viscozyme (a carbohydrase complex) have compared. Results showed that both enzymes increased the ratio of soluble to insoluble-bound phenolics, meaning more phenolics were converted into bioavailable forms. Moreover, viscozyme was more effective than Pronase, extracting higher amounts of gallic acid, catechin, and prodelphinidin dimer A, and uniquely releasing p-coumaric, caffeic acids, and procyanidin dimer B, which Pronase failed to extract. Furthermore, soluble phenolics from enzyme-treated samples significantly inhibited α-glucosidase and lipase, suggesting potential in managing hyperglycemia and obesity. Nevertheless, it has been reported that even after enzymatic treatment, some phenolics remained bound. Although EAE was effective, alkaline hydrolysis (NaOH) still achieved higher phenolic yields and antioxidant activity, indicating that EAE might need optimization to match chemical extraction efficiency.

Pulsed Electric Field (PEF) extraction is an emerging green technology that applies short, high-voltage pulses to create pores in cell membranes, a process known as electroporation. This increases cell permeability and facilitates the release of intracellular bioactive compounds. PEF enables efficient extraction at ambient temperatures, reducing both extraction time and energy consumption while preserving heat-sensitive compounds. It also requires smaller amounts of non-toxic solvents, minimizing environmental impact. However, optimization of field strength and process parameters is essential to balance extraction efficiency and prevent excessive cell damage [261]. In an extensive study, optimization of PEP parameters for recovering phenolic compounds from white grape pomace have been investigated [262]. Selected parameters including electric field strength, energy input, solvent composition, temperature, and extraction time were investigated. Study showed that optimal PEF parameters were 3.8 kV/cm, 10 kJ/kg, 50% ethanol, 50 °C, 190 min for recovering phenolic compounds of white grape pomace. Although, PEP provides higher yields, reduced solvent use, and shorter extraction times, further research on industrial validation, complete phenolic characterization, bioavailability testing, and sustainability assessment to unlock its full potential in the circular bioeconomy. Moreover, hybridizing green extraction methods such as integrating PEF with UAE, EAE, or MAE, can improve yields while lowering energy and solvent inputs. Overall, it has been highlighted that UAE, NADES, and UA-NADES offer better results in efficiency and green chemistry, while PLE provides higher yields in industrial application areas. Moreover, PEF and EAE are suggested as pretreatments to enhance other methods, while SFE provides pure extracts, especially in non-polar phenolic compounds. When used synergistically, combination of these methods has potential to provide high-value compounds for medical, pharmaceutical, cosmetic, and functional food applications [263,264,265].

Versatile wine production wastes, including grape pomace, seeds, and peels, have been investigated to analyze byproduct phenolic content, such as phenolic acids, stilbenes, and flavonoids [266]. They were studied to compare various techniques, namely, PEF, high-pressure processing, SFE, pressurized liquid, EAE, UAE, and MEA extraction methods. Results showed that MAE and UAE methods yielded the highest extraction and stronger antioxidant activity, while EAE method gave more purified extractions when it was combined with other methods. Moreover, the combination of UAE and PEF extraction methods preserved the total phenolic content by more than 40%. Since wine byproducts are not fully used, this study suggests that they can support zero-waste production and evaluate value-added recovery mostly in agriculture.

Natural deep eutectic solvent (NADES) extraction is a green extraction method that uses mixtures of natural compounds such as sugars, amino acids, and organic acids as eco-friendly solvents to extract bioactive compounds from plants or other materials. These solvents are formed by strong hydrogen bonding between components, creating a liquid with high solubility power. The method is efficient, safe, and biodegradable, making it suitable for food, cosmetic, and pharmaceutical applications. However, NADES are often viscous and difficult to recover, which can limit large-scale use [263,267]. In pursuit of a similar goal, Liu et al. [268] investigated the phenolic content of water caltrop shells, aquatic plants that are mainly distributed across Europe, Asia, and Africa, with a green extraction method named ultrasound-assisted natural deep eutectic solvents (UA-NADESs) to enhance the efficiency of extracting bioactive compounds from natural materials. Results showed a high yield phenolic compounds, which can be further investigated for applications in food preservation, cosmetic formulations, and pharmaceutical products. Correspondingly, rose oil production causes a lot of waste of phenolic compounds with high-yield byproducts. To recover them, the ultrafiltration method was studied using rose distillation wastewater [269]. Two membranes, 1 and 10 kDa, were tested, and 1 kDa showed higher purification and yield of phenolic compounds with 88.5%. Ultrafiltration pledges to a more isolated and stabilized recovery. Hence, these recovered compounds preserved their antioxidant activity and suggested that they can be further studied as cosmeceuticals, functional foods, natural preservatives, and nutraceuticals. Broccoli leaves are rich in oligosaccharides and bioactive compounds, including non-extractable phenolic compounds, giving them the potential for having functional properties [270,271]. To investigate bioactive properties and evaluate the functional dietary fiber SFE technology, which provides a higher concentration of oligosaccharides, improving water solubility and extraction yield of phenolic compounds to enhance antioxidant activity [272]. Results demonstrated that supercritical technology effectively valorizes broccoli leaf byproducts, evaluating their bioactive properties with optimal conditions of 191 bar, 40 °C, and 1 h. Citrus processing causes a wide range of citrus peel and waste products that are rich in various bioactive compounds, including phenolic content [273,274]. In a research article where lignocellulosic byproducts like STF231 that are derived from the medicinal plant extract industry have been used, different extraction techniques are compared to see the impact on their total phenolic content, as well as antioxidant activity [275]. In this study, conventional hydroethanolic extraction (CE), NADES, UAE, and their combinations are compared. CE is a classic method used to pull bioactive compounds out of plant material using a mixture of water and ethanol as solvent [276]. Results showed that CE exhibited the strongest antioxidant activity, while NADES and UAE combination produced the highest polyphenol recovery. Further analysis has been conducted using tools that focuses on evaluating the sustainability profile of the sample preparation phase, especially Green Analytical Procedure Index (GAPI), AGREE (Analytical Green Chemistry Evaluation), and AGREEprep (Analytical Green Chemistry Evaluation of Extraction Preparation) [277]. Analytical results showed that the combination of NADES and UAE methods were the most sustainable method, with low toxicity, low cost, and high efficiency, suggesting it as a sustainable alternative to traditional organic solvent extraction. It is also highlighted that only one specific plant byproduct was investigated in this case, indicating that results may vary across all species. Furthermore, no biological or in vivo tests were conducted to confirm health effects of the extracts.

Overall, it is notable to say that each technique offers advantages to be more suitable for specific applications while presenting divergent limitations. Conventional methods are still widely used, simple, and accessible, while suffering from higher solvent toxicity, poor economic sustainability, and lower extract quality and efficiency.

In comparison, SFE provides high extraction efficiency, better mass transfer, and is environmentally friendly while being a suitable alternative for thermolabile compounds. Nonetheless, it is a costly method with limited effectiveness for polar compounds. Unlike SFE, UAE is a cost-effective technique with a rapid and energy-efficient method with good performance at low temperatures. However, its efficiency depends on the sample, such as the plant matrix, and it may require additional steps like filtration. MAE also provides fast extraction with high yields, reduced solvent use, and good preservation of bioactive compounds. Nevertheless, it involves non-uniform heating, limited sustainability for non-polar compounds, and high equipment costs like SFE. EAE provides high selectivity and is suitable for mild operation conditions and release of bound phenolics. However, its high enzyme cost, high sensitivity to process conditions, and long extraction time are a challenge. PEF is an eco-friendly and highly efficient method that increases cell permeability and preserves heat-sensitive compounds. However, it involves high equipment costs, and it is less effective for highly rigid matrices. PLE provides high-efficiency extraction by using elevated temperature and pressure to enhance solvent solubility and penetration. This results in time-efficient extraction with reduced solvent consumption. However, it again requires high enzyme cost and has a risk of thermal degradation of thermolabile compounds. HWE, which is a safe, eco-friendly, and simpler method, is a suitable technique for polar compounds. Nonetheless, its major challenges are long extraction time, high thermal exposure, and risk of degradation of heat-sensitive compounds. Moreover, HC is an energy-efficient and solvent-saving technique that enhances mass transfer and extraction yields. Nonetheless, it has limited control over cavitation intensity and risk of degradation of sensitive bioactives. HPP is a high-efficiency method with high solvent access and minimal thermal damage, which makes this technique suitable for both polar and non-polar compounds. However, it requires high capital cost with complex process control. DESs, along with NADES, which is a type of DES, are emerging as green solvents with high extraction yields, but their high viscosity and the necessity of downstream filtration remain as challenges. Additionally, high-voltage electric discharge (HVED) is an energy-efficient method that provides high extraction yield. Nonetheless, it lacks selectivity and has a risk of causing compound degradation. Lastly, ohmic heating is an energy- and time-efficient method with reduced solvent usage, and it provides good preservation of heat-sensitive compounds. However, high equipment cost and limitation by dependence on the electrical conductivity of the material are the main challenges.

### 3.2. Food Industry

Date palm byproducts are produced by the processing of syrup production. To evaluate these low-cost agricultural wastes, functional biscuits have been aimed to be developed. Tests involved texture, color, proximate, sensory, and shelf life analysis [204]. Results demonstrated that total phenolic capacity and antioxidant activity have increased. Samples with more extracts showed hardness as texture compared to others, but water capacity remained stable across storage conditions. Moreover, biscuits with 10% date palm extract had the highest consumer acceptance with a balance in taste, texture, and nutritional enhancement. Hence, this study presents the date palm byproducts as functional foods with nutritional enhancement and sensory acceptability due to their higher dietary fiber, antioxidant, and anti-inflammatory properties. Phenolic compounds from date seeds and seed extracts exhibit strong antioxidant activities. Therefore, the use of date-based fibers or phenolic extracts has potential to improve the quality of foods. Moreover, they have potential for generating functional oils, natural additives. Although date seed powder is being used in food products, it has been highlighted that date seeds have not traditionally been consumed, suggesting more studies for safety profile. Food industry byproducts are rich in valuable phenolic compounds and no longer waste but strategic resources. Through green extraction techniques, including PLE, SFE, UAE, MAE, EAE, HC, and PEF, degradation of thermolabile compounds and recovery of phenolic compounds have been improved. Furthermore, through circular economy approaches, as well as less solvent usage and energy demand, these materials can be transformed into functional foods, nutraceuticals, active packaging materials, and biorefineries [278,279].

In another zero-waste valorization study, ginger peel extracts were studied for yogurt to test its efficiency for shelf life and microbiological stability [207]. Experiments were tested as fortification of yogurt with 0.5%, 1.0%, and 1.5% of ginger peel extracts for 21 days. Microbiological tests were performed as total viability count, formation of yeasts and molds, and activity of *L. bulgaricus* and *Streptococcus thermophilus* (*S. thermophilus*). Results showed that fortified yogurts with 1.5% ginger peel extracts enhanced antioxidant activity and phenolic content up to triple the amount of the control groups, which were yogurts with no extracts. Moreover, pH remained more stable, and water-holding capacity was increased. Color characteristics of fortified yogurts with more extracts exhibited darker colors due to higher phenolic content. According to results, 80% ethanol extracts exhibited highest efficiency. Moreover, freeze-drying process on samples along with 80% ethanol extraction resulted in the highest total phenolic content between samples. As for microbiological tests, both *S. thermophilus* and *L. bulgaricus* remained in the desired range, while yeast and mold formation were significantly suppressed, indicating a longer shelf life with various bioactive health benefits. In a similar aim, pomegranate peels were investigated with the microencapsulation method in vitro as jelly gummy prototypes to enhance their value as byproducts, since they are considered nearly 70% waste product from juice processing [208]. The polyphenol content of pomegranate peel shows an instability to oxygen, light, and heat, even though there is a high amount of phenolic content. To be able to eliminate this challenge, microencapsulation was performed to enhance stability and bioavailability. Results demonstrated that the microencapsulation method significantly enhanced their stability and bioavailability, being a sensory-acceptable and functional vehicle candidate as a valuable method for delivering extracts in high yields. Moreover, microencapsulated extract exhibited higher bioavailability but need for an optimization process. In another study, sweet orange peels were investigated by using plasma-activated water with a central composite rotatable design to eliminate compounds that reduce their nutritional values [209]. Experimental results showed that the total bioactive content was significantly enhanced by plasma-activated water, and there has been a reduction in unwanted compounds, including terpenoids, limonin, narginin, tannins, phytic acid, and saponins, suggesting plasma-activated water as a permissible method to eliminate undesirable compounds to evaluate nutritional value as nutraceutical or functional food candidates.

To evaluate the biological value and bioavailability of phenolic compounds of acacia bark and leaves, they have been investigated on raw ground beef patties to test their shelf-life development individually [211]. Results indicated that both phenolic content from leaves and bark significantly improved oxidative shelf life with higher antioxidant activity, reduced color deterioration, and lower lipid oxidation. In a comprehensive review about using phenolic compounds in meat products, it is suggested that nanotechnological methods, such as active films with polyphenols, improve their shelf life with more stability and antioxidant effect. Moreover, addressing challenges and limitations that affect phenolic compound efficiency has been listed as meat type, stabilization, dose optimization, standardization, and consumer accessibility [280].

In a comprehensive study, valorization of two agricultural byproducts, cheese whey and maize inflorescences, has been tested to investigate encapsulation efficiency of bioactive compounds such as phenolic compounds into lactose crystals, with the aim of enhancing their stability and antioxidant properties, as well as protecting them from degradation [212]. Results showed that lactose-polyphenol co-crystals that are developed from valorization of two byproducts provided antioxidant protection and stability under UV-light conditions with an efficient encapsulation capability, promising an industrial recovery of lactose and plant phenolic compounds. *Posidonia oceanica* is a widely studied seagrass that is endemic to the Mediterranean Sea. Billions of tons of biomass waste are obtained due to human activity and climate change [213]. To valorize these biomass wastes into sustainable byproducts, their bioactive properties have been investigated [281]. To make a comprehensive experiment, biomass wastes are prepared with three different methods: heat-assisted, UAE, and organic solvent-based. These extracts were tested on *Penicillium italicum* (*P. italicum*), *Botrytis cinerea* (*B. cinerea*), *Penicillium digitatum* (*P. digitatum*), *Penicillium expansum* (*P. expansum*), *Aspergillus niger* (*A. niger*), and *Geotrichum candidum* (*G. candidum*) to test their antifungal activity. Both UAE and heat-assisted extracts significantly inhibited *P. digitatum* and *B. cinerea*, while organic solvent-based extracts exhibited a general anti-fungal activity against all the targets. The same extract types were also tested on *murine norovirus* (MNV) and *feline calicivirus* (FCV). Results indicated that only organic-solvent-based extracts exhibited antiviral activity against FCV and MNV due to their lipid and non-glycosylated polyphenol content. Hence, it has been suggested that these biomass waste byproducts have a powerful value to be used in food preservation and biomedical applications. In an in vitro environmental sustainability study, blueberry fruit waste and its bioactivities were investigated in human keratinocytes HaCaT cells [214]. To extract the phenolic compound efficiently, optimized UAE was used with a response surface methodology. The extracted compounds exhibited high antioxidant activity due to their phenolic content, mainly anthocyanidins. Moreover, the antioxidant activity of these bioactive compounds preserved HaCaT cells from oxidative stress that is caused by hydrogen peroxide, suggesting a development for dietary supplements or functional food derivatives from blueberry pomace in the food industry.

### 3.3. Cosmetic Applications

Hazelnut byproducts are also abundant in bioactive compounds with a wide spectrum of properties. To evaluate the sustainability of hazelnut byproducts that were obtained from skin, shell, and defatted flour, NADES extraction has been used to compare traditional and UAE techniques [215]. Results showed that skin extracts contained a unique phenolic profile with the highest antioxidant activity compared to shell and defatted flour. Moreover, NADES with betaine and sorbitol yielded the highest efficiency for skin extracts, while the fructose and glycerol combination yielded the highest for defatted extracts and fructose and lactic acid for shell extracts. Compounds from hazelnut byproducts, such as catechins that are found in the skin extracts, protect against UV-induced damage and enhance photostability, while gallic acid and flavonols provide skin-healing properties due to their anti-inflammatory activity. Furthermore, the antioxidant activity of these extracts may prevent lipid peroxidation and aging progress, exhibiting anti-aging properties, making them valuable byproducts for health-related applications, anti-aging cosmetic products, and UV protection formulations.

Grape pomace flavonoids exhibit antimicrobial activities by chelating iron, forming hydrogen bonds between versatile microbial enzymes and proteins, protecting the structural integrity of Gram-positive bacterial cell walls, and disrupting the other membranes of Gram-negative bacteria [282]. Stilbenes from grape pomace have the potential to inhibit bacterial enzymes, namely, peroxidase, lactase, pectinases, xylanases, and cellulases, via degrading their tertiary structure [216,217]. Furthermore, antimicrobial activities against foodborne pathogens, namely *E. coli*, *S. Typhimurium*, *S. aureus*, *P. aeruginosa*, and *L. monocytogenes*, have also been demonstrated [228]. When phenolic compounds from grape pomace are combined with antibiotics, such as β-lactams, amphenicols, tetracyclines, and quinolones, a synergistic effect of inhibition against *S. aureus* and *E. coli* has been observed [216].

Phenolic compounds from date seeds and seed extracts exhibit strong antioxidant activities. Therefore, use date-based fibers or phenolic extracts to improve the quality of foods. Moreover, they have potential for generating functional oils and natural additives. Although date seed powder is being used in food products, it has been highlighted that date seeds have not traditionally been consumed, suggesting more studies for safety profile [220]. Moreover, it has been mentioned that green extraction methods can be a suitable alternative for their cost-effective and target-selective properties. Since olive and wine byproducts are rich in phenolic compounds, especially hydroxytyrosol, tyrosol, oleuropein, caffeic acid, rutin, catechin, and resveratrol, they are valuable candidates for sustainable biomass waste sources. These recovered phenolic compounds can be applied as natural antioxidants in foods, oil, and beverages, as well as nutraceuticals and supplements for health benefits due to their bioactivities. Moreover, they have been suggested as potential candidates as cosmeceuticals due to their UV-protective and anti-aging properties. Nevertheless, it has been highlighted that many green extraction methods have stability issues and the initial equipment and operating costs are high for supercritical and membrane systems [221,222,223]. As mentioned, grape byproducts are rich in phenolic content, dietary fiber, and other bioactive compounds [283]. Phenolic compounds, including catechins, resveratrol, and gallic acid, significantly exhibit antimicrobial activity against *S. epidermidis*, *E. coli*, and *S. aureus* via disrupting bacterial cell walls [283,284]. Moreover, evidential studies that experimented with grape byproducts in the Kirby–Bauer method demonstrated that they exhibit inhibition of biofilm formation of Gram-positive bacterial zones, significantly against *Cutibacterium acnes* [218]. These antimicrobial properties of grape byproducts provide stability for products, promising candidates for acne-prone skin, protection from outer contamination, and reduced inflammation and redness, making them suitable for integration into nutraceuticals and cosmetic products [219].

### 3.4. Pharmaceutical and Health Applications

Correspondingly to the previously discussed study, activities of bounded polyphenols from persimmon byproducts were investigated to contribute them as food byproducts, prebiotics, therapeutics, or nutraceutical agents [226,227]. Results showed dietary fibers selectively inhibited *E. coli* and biofilm activity, along with synergistic bactericidal effects with gentamicin (an aminoglycoside antibiotic) against *S. aureus*. Moreover, it inhibited virulence factors by inducing membrane damage, inhibiting biofilm formation, and blocking β-lactamase that induces multi-resistance to the β-lactam antibiotics. Furthermore, it has also been observed that they inhibited pathogen adhesion to HaCaT and Caco-2 cells, making these persimmon-derived phenolic compounds, significantly gallic acid and quercetin, powerful candidates for being used as nutraceuticals or functional ingredients to mitigate various bacterial infections.

Cacao bean shells are rich in phenolic content. In a recent study, cacao bean shell extracts from different origins were tested for their phytochemical profile and antimicrobial activity [224]. Results showed that extracts exhibited antimicrobial activity against *Streptococcus mutans* (*S. mutans*). Moreover, analyses confirmed one metabolite, 7-methylxanthine; however, it did not show an inhibitory effect against *S. mutans* when it was tested alone, suggesting that a synergistic effect with other extracts might give a potential activity. Grape pomace is rich in bioactive compounds, including versatile phenolic compounds, and it has been shown that these compounds improve lipid profiles by affecting oxidized low-density lipoproteins and lipid serums, affecting cardiovascular diseases [228].

Since traditional solvents like ethanol, acetone, and methanol are effective but toxic to the environment, Wang et al. studied recovering these phenolic compounds from citrus peels by using a green method of integrated extraction–adsorption system using resins and deep eutectic solvent (DES), which is less toxic and more biodegradable [274,285]. Results of the usage of the DES extraction–resin adsorption system showed a significant yield of recovery for phenolic compounds. Moreover, extracted phenolic compounds preserved their antioxidant and α-glucosidase inhibitory activity, promising to be strong candidates for natural agents for type 2 diabetes. In an in vitro study, mango peel extract was investigated for its photochemical profile in cultured cell lines, namely Vero cells and cancerous HepG-2 liver cells [229]. More than 15 phenolic compounds were identified, while gallic acid and chlorogenic acids were found abundantly. Identified compounds exhibited antimicrobial activity against *S. aureus and S. mutans*, antiviral activity against adenovirus-7 and HSV-1, and cytotoxicity against HepG-2 liver cancer cells, suggesting their use as a therapeutic agent in oncology and infectious disease management.

Avocado seed is considered one of the most highly valued fruits in the world, with various components including vitamins, enzymes, proteins, oligosaccharides, and phenolic compounds [286]. Industrial processing of avocado has resulted in a substantial amount of waste from seeds and peels due to a lack of information on recovery methods. To open a gate to eliminate this problem, many studies have been conducted, and one of them analyzed phenolic content of avocado seeds to evaluate its nutritional value in agriculture. Results showed that avocado seed powder consists of high tannins and flavonoids like quercetin and gallic acids [230]. Moreover, antimicrobial activities of avocado seed polyphenols have been documented against *E. coli*, *S. aureus, Salmonella* spp., *P. aeruginosa*, and *C. albicans* via disrupting bacterial cell walls, inhibiting bacterial enzymes, and targeting fungal pathogens. Furthermore, anti-inflammatory activity against pathways like TNF-α, IL-6, and IL-1β, and COX-2 and iNOS enzymes that are crucial for the inflammation process have been observed, promising a powerful candidate for functional food additives. Polyphenolic compounds of cherry byproducts from seeds are abundantly identified as caffeic, ferulic, gallic, and protocatechuic acids. These compounds modulate insulin sensitivity and lipid metabolism and enhance glucose utilization, which are key metabolic pathways of metabolic syndrome [231,232]. Additionally, they can contribute to glucose intake by modulating GLUT4 by influencing its expression and translocation [233]. Longan fruit contains valuable bioactive compounds, including proanthocyanidins, alkaloids, and phenolic acids, that provide benefits for managing non-alcoholic fatty liver disease (NAFLD) and obesity. Byproducts of longan fruit have been investigated for their antioxidant and anti-inflammatory activities. Slides demonstrated that they can regulate lipid metabolism genes and inflammatory pathways, enhance liver detoxification, and show potential for liver protection. Additionally, exhibiting potent activities makes them outperform synthetic antioxidants and makes them potential candidates for a more sustainable option for health-related applications [234].

In a recent in vivo study, both extractable and non-extractable polyphenols are investigated for their potential activity against obesity and metabolic disorders [235]. The treatment of obese rats with extractable and non-extractable polyphenols was conducted over a period of 8 weeks. Results demonstrated that non-extractable polyphenols exhibited better results by decreasing liver triglycerides by over 45%. In contrast, no reduction in insulin resistance from either type of polyphenol was observed. Walnuts have been used for their versatile benefits and put through various processing, such as oil production. The byproduct of the walnut oil process, walnut kernel with pellicle (WKP), has been investigated for its effect on gastrointestinal digestion via valorization of protein- and polyphenol-rich hydrolysates.

Further study investigated the anti-aging activity of WKP hydrolysates in vivo by testing d-galactose-induced aging mice. Experimental results demonstrated that WKP hydrolysates restored total antioxidant capacity and superoxide dismutase (SOD) activity, tissue morphology in liver and kidney, and reduced concentration of malondialdehyde (MDA), a byproduct of lipid peroxidation, in liver, kidney, and serum tissues. Thus, WKP hydrolysates are potential candidates as reusable byproducts with health benefits and sustainable properties. Hemp leaf byproducts contain a high amount of cannabinoids, flavonoids, etc., which shows a variety of these bioactive compounds in abundance in seeds, stems, hurds, and leaves themselves [287,288].

An in vitro study investigated these hemp leaf byproducts with hyaluronic acid gel formulations, which are a commonly used gel type for medical applications, such as for viscous supplementation for joint disorders [236]. Experimental studies are conducted in macrophage-like (RAW 264.7) and chondrocyte-like (C-20/A4) cell lines. Results showed that hyaluronic acid gel provided a sustainable and localized delivery of cannabinoids to joint spaces, reduced symptoms of conditions like osteoarthritis, and improved joint function by restoring viscoelastic properties of synovial fluid, promising a powerful candidate for therapeutic applications. The bark of the pomegranate tree (*Punica granatum*) is enriched with versatile bioactive compounds, namely phytochemicals like flavonoids, phenolic acids, and tannins, which are considered agro-industrial waste [289]. To evaluate its agricultural value, its antidiabetic properties were investigated in vivo [237]. In this study, the Soxhlet extraction method has been used to evaluate extraction efficiency and to reduce the risk of contamination with unwanted compounds with continuous recovery of analytes of the sample matrix. The results demonstrated that extracts evaluated inhibitory effects on α-glucosidase and α-amylase, exhibiting significant enzyme inhibition with no acute toxicity in mice at 2000 mg/kg. Garlic byproducts consist of proteins, phenolic compounds, dietary fibers, and polysaccharides, and they can be generated from harvesting or processing, such as stems, skin, leaves, and root plates [290]. Qui et al. [238] investigated garlic byproducts for their antioxidant, antimicrobial, and anti-inflammatory properties to find sustainable valorization methods. The comprehensive investigation suggested that challenges like a lack of standardization and material assessment and the necessity of cost-effective green methods with analysis need to be addressed to valorize garlic byproducts for potential applications in material, health, and agriculture-based applications. Bioavailability is one of the other challenges in this application area because phenolic compounds are generally poorly absorbed, rapidly metabolized, and present at very low aglycone levels in plasma. By designing synergistic polyphenol combinations, formulations to improve oral take and consider interactions with drugs that affect pharmacokinetics, and enhanced bioavailability, as well as their role in application areas such as in food industry, pharma, medical, and cosmetics [291].

### 3.5. Sustainable Materials and Packaging

In a 2024 research article, antioxidant activities of peel and seed extracts from mango, papaya, and loquat using different extraction solvents have been investigated to analyze and evaluate the potential antioxidant effects of these extracts in cooked chicken models during refrigerated storage [240]. During investigation, the study focused on their ability to control lipid and protein oxidation and maintain color stability. Experimental results showed that regardless of solvent, mango seed extracts exhibited the strongest antioxidant activity in ABTS, DPPH, and FRAP assays. Furthermore, mango seed extracts had the highest levels of phenolic compounds, flavonoids, and condensed tannins. It has also been pointed out that seed extracts were richer in antioxidants than peel extracts. Applicational results highlighted that mango seed extracts maintained color better and effectively reduced lipid and protein oxidation during ten days of refrigerated storage. Papaya and loquat extract also reduced oxidation in a less effective way. The study suggested that evaluating the effects in different meat types and processing conditions can be further investigated for future approaches. A 2019 review discussed several future approaches to optimize process, assess sustainability, and improve stability [292]. For instance, combining processes to extract multiple compounds from same waste stream to maximize value, applying response surface methodology or machine learning models to optimize extraction parameters have been discussed. Moreover, it has been suggested that using nano- and microencapsulation can improve stability and controlled release of sensitive bioactives in food or nutraceutical applications. Further limitations can be listed as complication in collecting, processing, and standardizing raw byproducts that have short shelf life because of high moisture content [220,293]. Biodegradation refers to the natural breakdown of materials through enzymatic processes initiated by microorganisms. To improve functional properties, the combination of bioplastics with complementary barrier capabilities has been investigated using polymer blending and multilayer configurations [35]. Results indicate phenolic acids are a powerful candidate for food packaging and preservation applications due to their broad spectrum of antioxidant, antimicrobial, and antiviral activities. To evaluate one of the most valuable waste products into valuable byproducts, Fernandes et al. [241] investigated forest byproducts for developing sustainable particleboards to potentially contribute to the food-packaging industry. In these experiments, poplar bark and veneers with bio-adhesives containing citric acid have been used. Experimental studies indicated that citric acid enhanced the bonding properties by more than 50% but negatively stimulated the antioxidant capacity. Thus, particle boards exhibited high mechanical resistance and antioxidant activity, making them a potential candidate for eco-friendly, sustainable food packaging.

Overall, green extraction techniques have a promising potential as an alternative to conventional methods. Nonetheless, their broader industrial application areas require careful consideration of several key challenges as discussed previously. To summarize these challenges, standardization of processing parameters remains an essential aspect to ensure reproducibility, along with consistent extraction efficiency and reliable dose determination of bioactive compounds generally. Additionally, standardization of extracts is necessary for downstream applications, including encapsulation or formulation optimization or in vitro and in vivo applications. Moreover, enhancing extraction efficiency in terms of time and yield with reduced solvent consumption is essential for cost-effective and suitable production. Although most green extraction techniques demonstrate high yields with short process times, further optimization is required for energy input, solvent selection, and scalability. By addressing these limitations, their enhanced potential will expand their application areas, including the food, pharmaceutical, cosmetic, health, and sustainable food packaging industries. It is notable to say that a strategic combination of suitable green extraction methods offers a potential pathway to overcome current challenges. Byproducts are emerging for their significant economical and environmental advantages. Hence, overcoming these challenges and improving extract quality and bioavailability will enable the recovery of higher-value bioactive compounds and facilitate the improvement toward more suitable and industrially viable extraction processes.

## 4. Conclusions

Phenolic compounds form byproducts that possess beneficial attributes with their immense biological properties, including antioxidant, antimicrobial, antiviral, anti-inflammatory, and antifungal activities. Valorization of these phenolic compounds from waste products requires unique methods that are also acquiring eco-friendly and cost-effective properties. To overcome limitations such as standardization, bioavailability, sustainability, dose determination, delivery, stability, lack of variability management, and knowledge, innovative methods need to be developed. Several extraction techniques, including HC, hot-water extraction, SFE, PLE, UEA, EAE, MAE, PEF, high-pressure processing extraction, and NADES-based techniques have been indicated to have the potential to overcome some of the challenges during sustainability studies, but additional research is required. The main challenge remains scaling sustainable and optimized processes while ensuring economic and regulatory practicability, leading to zero-waste and high valuable food systems. Moreover, assessing synergistic or antagonistic interactions between different byproduct materials, as well as investigating encapsulation or formulation strategies, is essential to improve extract stability and application in the food industry, as well as in other application areas. Furthermore, there is insufficient standardization in phenolic extraction, identification, and quantification. These different methods produce incomparable data, reducing reliability, even though they are sustainable and powerful candidates for green extraction methods. Even though some of the byproducts, including pomegranate peel, ginger peel, and longan fruit waste extracts, are well studied, further research is needed for some of the byproducts, including avocado seed, coffee, and seagrass, to uncover their efficiency in their potential application fields. Although byproducts from phenolic compounds are risen interest, other than TPC, their activity against specific molecules or interactions such as polyphenol–polyphenol or polyphenol–protein, are merely discussed. Furthermore, in vivo studies are still limited and well-designed clinical trials using polyphenol-rich byproducts extracts as bioactives are poorly defined. Similarly, the majority of in vitro studies demonstrate only in vitro activity and perform further studies like dose-dependent modeling subtly. Moreover, even though green extraction methods provide many advantages, processes in whole extracts are still limited compared to purified standards. As previously mentioned in antimicrobial section, different processes and methods make it difficult to directly compare values, therefore translating byproduct phenolic compounds into real antimicrobial products. This gap also makes it difficult to understand about selectivity of byproduct phenolic compounds. Moreover, while phenolic compounds are generally seen as safe and natural, more studies about pro-oxidant, genotoxic, or endocrine effects at certain doses or in certain forms for byproduct extracts. The food industry and agricultural applications have been rapidly evolving, but further studies and information need to be gathered to equally grow the evolution in cosmetic and health-related applications. Warranting further research will potentially help to evaluate the food and meat industry, along with cosmetic, biomedical, health-related, and agricultural applications, and evolve the sustainable research to overcome limitations in methods, knowledge, underusage, bioavailability, and nutritional value of phenolic compounds from byproducts.

## Figures and Tables

**Table 1:** Overview of industrial application areas and experimental contexts for phenolic compound byproducts, including their sources and application outcomes.

Industrial Application Area	Experiment Area	Phenolic Compound Byproduct Source	Type of Phenolic Compound	Type of Extraction Method	Bioactivity and Results of Applications	Reference
Food Industry	Syrup production Food additives in biscuits	Date palm and seed extract Date seed powder	Flavonoids Anthocyanins	Microwave-assisted extraction method	Increase in antioxidant activity The dose of sample affected the texture as hardness Balance in taste and texture Enhancement in nutritional value	[204,205]
	Fortification in yogurt (0.5%, 1.0%, and 1.5%)	Ginger peel extracts	Gingerol Anthocyanins Flavonoids	Ultrasound-assisted extraction Conventional solvent extraction using hot water, 80% ethanol, and 100% ethanol	Yogurt samples that are fortified with 1.5% ginger peel extracts enhanced the antioxidant activity Increase in water-holding capacity Increase in stability of pH Fortified yogurts exhibited darker colors due to higher phenolic content Microbiological tests that were carried out with *S. thermophilus* and *L. bulgaricus* resulted in longer shelf life potential with suppressed yeast and mold formation	[206,207]
	Jelly gummy	Pomegranate peel extracts	Punicalagin (both α and β form) Ellagic acid Gallic acid Coumaric acid Catechin Protocatechuic acid Chlorogenic acid Epicatechin Caffeic acid 4-Hydroxybenzaldehyde Benzoic acid Ferulic acid Quercetin	Conventional solid–liquid extraction	Bioavailability enhancement Increased firmness in texture Similar hardness between samples	[208]
	Investigation of bioavailability enhancement using plasma-activated water method	Sweet orange peels	Benzoic acid Ferulic acid Caffeic acid Myricetin 3,5,6,7,4′-pentamethoxyflavone 3,5,6,7,3′,4′-hexamethoxyflavone Naringin Protocatechuic acid Apigenin Naringenin Hesperidin Hesperetin Flavonoids Narirutin Tannins Sinapinic acid Isovanillic acid 4-coumaric acid Tangeretin Neohesperidin	Plasma-activated water method	Decrease in unwanted compounds, which increased the bioavailability	[209,210]
	Raw ground beef patties	Acacia bark and leaves	Flavonols, Proanthocyanidins Bioflavonoids/polyflavonoids Hydrolyzable tannins Hydroxybenzoic acids Hydroxycinnamic acids Dihydrochalcones Abundantly: Epicatechin p-coumaroyltrifolin B Procyanidin B5 (−)-epigallocatechin Procyanidin C1	Conventional solid–liquid extraction	Both samples exhibited an increase in shelf life and stability Higher antioxidant capacity Reduction in color deterioration Reduction in lipid peroxidation	[211]
	Investigation of encapsulation efficiency with lactose crystals	Cheese whey and maize inflorescences	Chlorogenic acid Maysin	Hot-aqueous extraction and encapsulation	Increasement in antioxidant capacity, stability, and bioavailability Protection from degradation	[212]
	Investigation of effect in bioavailability	*Posidonia oceanica* (seagrass)	Flavonoids Stilbenes Coumarins Tannins Lignans	Solid–liquid extraction	Antifungal activity against *Penicillium italicum*, *Botrytis cinerea*, *Penicillium digitatum*, *Penicillium expansum*, *Aspergillus niger*, and *Geotrichum candidum* Antiviral activity against *murine norovirus* and *feline calicivirus* Potential in food preservation	[213]
	Human keratinocytes HaCaT cells	Blueberry pomace	Cyanidin-3-glucoside Quercetin-3-glucoside	Ultrasound-assisted extraction	High antioxidant activity Preservation of HaCaT cells from oxidative stress Developmental potential as food additives	[214]
Cosmetic Applications	Investigation with NADES extraction process to enhance bioavailability	Hazelnut skin and shell	(−)-epicatechin (+)-catechin Phloridzin Quercetin-3-*O*-rhamnoside Myricetin-3-*O*-rhamnoside	NADES extraction	Protection against UV-induced damage Enhancement in photostability Antioxidant and skin-healing properties Promoter in process of inhibition of lipid peroxidation and the aging process	[215]
	Investigation with antibiotics (β-lactams, amphenicols, tetracyclines, and quinolones)	Grape pomace	Catechins Resveratrol Gallic acid	Solid–liquid extraction Supercritical Fluid extraction Ultrasound-assisted extraction Microwave-assisted extraction Enzyme-assisted High-Voltage Electric Discharge Ohmic Heating High-Pressure Processing Deep Eutectic Solvents (DESs)	Inhibition of antibacterial enzymes, including peroxidase, lactase, pectinases, xylanases, and cellulases Antimicrobial properties against *E. coli*, *S. Typhimurium*, *S. aureus*, *P. aeruginosa,* and *L. monocytogenes* Synergistic effect on the inhibition of *S. aureus* and *E. coli* Potential as cosmeceuticals and nutraceuticals by inhibiting *Cutibacterium acnes*, *as well as reducing inflammation and redness*	[216,217,218,219]
	Investigation of their effect on food quality and methodology efficiency	Date seed powder	Protocatechuic acid p-hydroxybenzoic acid Vanillic acid Syringic acid Caffeic acid Coumaric acid Ferulic acid p-hydroxycinnamic acid Chlorogenic acid Quercetin-3*-O*-glucoside Catechin Procyanidin Anthocyanins Proanthocyanidins	Conventional solvent extraction	Strong antioxidant activity Potential to enhance food quality as food additives and functional oils	[220]
	Investigation into bioavailability and potential applications	Olive byproducts	Hydroxytyrosol Tyrosol Oleuropein Caffeic acid Rutin Resveratrol Catechins	Ultrasound-assisted extraction Microwave-assisted extraction Enzyme-assisted extraction Supercritical Fluid extraction Pressurized Liquid extraction Ohmic Heating Pulsed Electrical Field	Potential as natural antioxidants in food, oil, and beverages Sustainable biomass waste sources	[221,222]
	Investigation into bioavailability and potential applications	Wine byproducts	Hydroxytyrosol Tyrosol Oleuropein Caffeic acid Rutin Resveratrol Catechins	Ultrasound-assisted extraction Microwave-assisted extraction Enzyme-assisted extraction Supercritical Fluid extraction Pressurized Liquid extraction Ohmic Heating Pulsed Electrical Field	UV-protectant and anti-aging properties Sustainable biomass waste sources Natural antioxidants	[221,223]
Pharmaceutical and Health Applications	Investigation on bioactivity	Cacao bean shells	Quercetin Kaempferol Proanthocyanidins	Microwave-assisted extraction Ultrasound-assisted extraction	Rich phenolic content Antimicrobial activity against *S. mutans* 7-methylxanthine metabolite did not exhibit inhibitory effect against *S. mutans* alone	[224,225]
	Investigation into bioactivity and potential health applications	Persimmon byproducts	Gallic acid Quercetin	Solid-assisted extraction	Antimicrobial activities: Inhibition of *E. coli* and biofilm activity Synergistic effect with gentamicin against *S. aureus* Potential candidate by inhibiting bacterial adhesion to HaCaT and Caco-2 cells Antiviral activity by inhibiting biofilm formation, blocking β-lactamase	[226,227]
	Investigation of bioactivity and effect of using a DES extraction–resin adsorption system	Grape pomace	Quercetin-3-*O*-rutinoside Quercetin-*O*-pentoside Myricetin-*O*-rutinoside Gallic acid Caffeic acid Syringic acid Vanillic acid Chlorogenic acid *p*-coumaric acid Petunidin-rutinoside Malvidin-rutinoside β-Type (epi)catechin tetramer Quercetin-glucuronide *p*-coumaric acid Malvidin-3-*O*-glucoside Malvidin-3-*O*-(*p*-coumaroyl)glucoside (+)-catechin (−)-epicatechin Syringic acid (−)-gallocatechin	Solid–liquid extraction Supercritical Fluid extraction Ultrasound-assisted extraction Microwave-assisted extraction Enzyme-assisted High-Voltage Electric Discharge Ohmic Heating High-Pressure Processing Deep Eutectic Solvents (DESs)	Improving lipid profiles that are effective for cardiovascular diseases Being able to preserve their antioxidant and α-glucosidase with DES extraction–resin adsorption system	[228]
	In vitro study with Vero cells and HepG-2 liver cells	Mango peel extract	Gallic acid Chlorogenic acid	*	Antimicrobial activity against *S. aureus and S. mutans* Antiviral activity adenovirus-7 and HSV-1 Cytotoxicity effect against HepG-2 liver cancer cells	[229]
	Investigation into bioactivity	Avocado seed powder	Quercetin Gallic acid	Conventional solid–liquid extraction	Antimicrobial activity against *E. coli, S. aureus, Salmonella* spp., *P. aeruginosa,* and *C. albicans* Anti-inflammatory activity against TNF-α, IL-6, and IL-1β pathways and COX-2 and iNOS enzymes	[230]
	Investigation into bioactivity	Cherry seed	Caffeic acid Ferulic acid Gallic acid Protocatechuic acid	Conventional solid–liquid extraction Ultrasound-assisted extraction Enzyme-assisted extraction Supercritical Fluid extraction	Modulation of lipid metabolism and insulin sensitivity Promote glucose utilization Contribution to glucose intake	[231,232,233]
	Investigation into bioactivity	Longan fruit	Proanthocyanidins Phenolic acids	Ultrasound-assisted extraction Enzyme-assisted extraction Microwave-assisted extraction	High antioxidant and anti-inflammatory activities Potential candidates against NAFD and obesity	[234]
	In vivo experiment with d-galactose-induced aging mice	Walnut oil process byproducts	Procyanidin dimer B2 isomer I Procyanidin dimer B1 isomer II Malvidin hexoside Malvidin Coumaroyl-hexoside Petunidin hexoside (−)-epicatechin Quercetin Glucuronide Myricetin Rhamnoside (+)-catechin (iso)-rhamnetin Myricetin	Conventional solid–liquid extraction	Restore in antioxidant and SOD activity with WKP hydrolysates Improvement in liver and kidney tissue morphology Reduction in MDA concentration Reduction in expression of SREBP-1c by pretreatment of malvidin hexosides	[235]
	In vitro study to investigate the potential in joint disorders using macrophage- and chondrocyte-like cell lines	Hemp leaf	Cannabinoids Flavonoids	Heat-reflux extraction	Reduction in symptoms of osteoarthritis Improvement in joint function	[236]
	In vivo study for investigation of their antidiabetic activity	Pomegranate tree (*Punica granatum*) bark	Flavonoids Phenolic acids Tannins	Soxhlet extraction	Inhibition of α-glucosidase and α-amylase	[237]
	Investigation into sustainable methods to evaluate bioactivity	Garlic byproducts	Flavonoids	Supercritical Fluid extraction	No significant improvement observed Lack of standardization and material assessment	[238,239]
Sustainable Materials and Packaging	Investigation into their bioactivities in cooked chicken noodle	Mango, papaya and loquat byproducts	- In mango: Mangiferin Catechin Quercetin Kaempferol Rhamnetin Anthocyanins Gallic acid Ellagic acid Propyl gallate Methyl gallate Benzoic acid Protocatechuic acid - In loquat: 3-p-coumaroylquinic acid Caffeoylquinic acid 5-feruloylquinic acid Quercetin Kaempferol derivatives, including galactoside, glucoside, and rhamnoside - In papaya: Ferulic acid Mandelic acid Syringic acid Vanillic acid Myricetin Conifer aldehyde	Conventional solid–liquid extraction	Strongest antioxidant activity in mango seed extracts Higher phenolic content in seed extracts than peel extracts Better color maintenance in mango seed extracts Reduction in lipid and protein oxidation in mango peel extracts Partial effect of papaya and loquat extracts in reduction in lipid and protein oxidation	[240]
	Investigation into their potential in food-packaging industry using polyphenols and citric acid	Poplar bark and veneer	*	Chemical-assisted solid–liquid extraction	Resulted in high antioxidant activity, mechanical resistance, and bonding properties	[241]

* Not identified.

## Data Availability

No new data were created or analyzed in this study. Data sharing is not applicable to this article.
